# Modeling Neuroimmunological Interactions at the Blood–Brain Barrier Using In Vitro 3D Human Organoids: Inflammation and Ischemia–Reperfusion Injury

**DOI:** 10.3390/cells15131173

**Published:** 2026-06-27

**Authors:** Aya A. Eltaibany, Kathleen McGovern, Goodwell Nzou, Daniel Porada, Michael C. Seeds, Anthony Atala

**Affiliations:** Wake Forest Institute for Regenerative Medicine, Wake Forest School of Medicine, Winston-Salem, NC 27101, USA; katgov305@gmail.com (K.M.); goodwell.nzou@advocatehealth.org (G.N.); daniel.porada@wfusm.edu (D.P.); michael.seeds@advocatehealth.org (M.C.S.); anthony.atala@advocatehealth.org (A.A.)

**Keywords:** blood–brain barrier, immune cells, neuroimmune, ischemia–reperfusion, inflammation, cell adhesion molecules

## Abstract

**Highlights:**

**What are the main findings?**

- We validated the feasibility of using a 3D self-assembled blood–brain barrier (BBB) model compromised of different brain cell types, to study BBB disruption induced by both inflammation (TNF-α, IFN-γ) and ischemia–reperfusion injury (oxygen-glucose deprivation-reoxygenation model, OGD-R). Organoids showed increased permeability to fluorescent dextrans, decreased tight-junction gene expression, elevated ROS/hypoxia signals, and upregulated cell adhesion molecules such as ICAM1 and VCAM1.
- We demonstrated the ability to detect changes in immune cell transmigration across the BBB into the organoids under both conditions of inflammation and OGD-R. Increased immune cell infiltration after cytokine treatment and reperfusion was blocked by anti-cell adhesion molecule antibodies.

**What are the implications of the main findings?**

- This 3D human BBB organoid provides a biologically relevant platform for modeling neuroimmune interactions, enabling studying how inflammation and ischemia–reperfusion injury alters barrier integrity, adhesion molecule expression, and immune cell entry in a human-specific context.
- The model offers a powerful predictive preclinical screening tool that bridges the gap between simple, physiologically irrelevant 2D models and expensive in vivo animal models and enables high throughput screening of various anti-cells adhesion molecule therapies. In vitro model representative of human tissue complexity accelerates the translation of drugs into clinical practice.

**Abstract:**

Numerous central nervous system pathological conditions involve blood–brain barrier (BBB) disruption and the egress of immune cells in the brain. Controlling immune cell transmigration into the brain represents a potential therapeutic target. This study describes the application of a 3D human BBB spheroidal model that consists of six major brain cell types to test the transmigration of immune cells under normal and pathological conditions of inflammation and ischemia–reperfusion injury (IRI). The cell types in the BBB organoid include brain microvascular endothelial cells (HBMVECs) and pericytes at the spheroids’ surface, surrounding a core of astrocytes, microglia, oligodendrocytes, and neural progenitor cells. The model recapitulates the interaction of CD4^+^ T-cells and immunomodulators with HBMVECs at the BBB including changes in cell adhesion molecules expressed on their surface. This study demonstrated that the human 3D BBB model recapitulates many features of the barrier under normal and pathological conditions of inflammation and hypoxia-reperfusion injury. Proinflammatory cytokines and hypoxia disrupt the barrier and increase its permeability, decreasing the expression of tight junctions. Proinflammatory cytokines and reperfusion increase the expression of cell adhesion molecules and increase immune cell transmigration. Immune cell transmigration could be reduced with anti-cell adhesion molecule antibodies, further validating the model for studying neuroimmune interactions and for conducting high-throughput screening of therapeutic targets that modulate immune cell transmigration into the brain.

## 1. Introduction

A large spectrum of neurological disorders such as multiple sclerosis, neuromyelitis optica spectrum disorders, Alzheimer’s disease [1], epilepsy [2], and reperfusion injury after ischemic stroke share a degree of blood–brain barrier (BBB) disruption that results in immune cell infiltration into the brain to various extents [3]. The BBB is the dynamic interface formed by brain microvascular endothelial cells (BMVECs) to protect the brain’s homeostasis. It also regulates the passage of immune cells from peripheral circulation into the brain. Even though BMVECs are the principal structural components of the BBB, other cellular components such as pericytes/vascular smooth muscle cells, astrocytic end-feet, glial cells and basement membranes all participate in the formation of the barrier [4].

BMVECs regulate brain homeostasis through the presence of tightly sealed paracellular junctions, the absence of fenestrations, and decreased pinocytic activity. BMVECs also possess a variety of active influx transporters for essential nutrient molecules and different efflux transporters that pump toxic compounds out into the circulation [5]. Migration of immune cells into the brain is a highly regulated series of interactions between the immune cells and BMVECs. Cell adhesion molecules expressed on the BMVECs contribute to these interactions by binding to their complementary ligands on the surface of activated immune cells. Two well-studied adhesion molecules are intercellular adhesion molecule 1 (ICAM1) and vascular cell adhesion molecule 1 (VCAM1), which bind to their respective ligands, α1β2 [lymphocyte function-associated antigen 1 (LFA-1)], and α4β1 [very late antigen 4 (VLA-4)] integrins, which are upregulated on CD4^+^ T cells during inflammation [6].

The properties of the BMVECs and the elevated level of regulation found at the BBB interface that control interactions with immune cells are not intrinsic to microvascular endothelial cells (BMVECs) but emerge through their assembly and interactions with other cells of the neurovascular unit (NVU) [7]. This NVU consists of BMVECS lining the luminal sides of the vessel in the brain, pericytes, and astrocytes in close contact on the abluminal surface. Neurons, microglia, and oligodendrocytes are also involved in the generation and regulation of BBB functional integrity [8,9]. The detailed contribution of each cell type to the formation of the BBB is beyond the scope of this article but readers are referred to more comprehensive reviews of such regulation [10,11].

Unlike the quiescent state of the BBB that limits the passage of molecules and transmigration of immune cells into the brain [12,13], various neurological disorders affect the integrity of the BBB and show upregulation of cell adhesion molecules on the BBB surface. This upregulation enables immune cells to migrate into the CNS parenchyma initiating or exacerbating disease conditions. These disorders would benefit from therapeutic strategies that modulate or block cell adhesion molecules. A clinically successful example is Natalizumab, an anti-α4 integrin monoclonal antibody (mAb) that blocks adhesion between T cells and VCAM1 on the surface of endothelial cells and was the first FDA-approved anti-cell adhesion molecule mAb for the treatment of relapsing multiple sclerosis (MS). Natalizumab has shown greater efficacy than other drugs used for MS [14] and is still one of the most effective drugs against the formation of new lesions initiated by immune cell migration [15]. Anti-cell adhesion molecule therapy has also shown some encouraging therapeutic potential in other neurological disorders, including Alzheimer’s disease [1], epilepsy [16], stroke, and associated reperfusion injury [17].

Despite the encouraging results of anti-cell adhesion molecule therapy in the treatment of MS and other disorders, few novel therapeutic targets exist that are specific to the brain. Most in vitro transmigration studies have been conducted on transwell BBB models, often using nonhuman cells in a monoculture of BMVECs [18]. Failure to translate drugs into clinical practice is attributed in part to the absence of 3-dimensional (3D) physiological models that can recapitulate the physiology of the specific human organ of interest [19].

We have successfully created 3D BBB NVU tissue organoids composed of six human brain cell types using a core of human iPSC-derived neuroprogenitor cells, microglia, astrocytes, and oligodendrocytes with an external surface composed of primary endothelial cells and pericytes [20,21,22]. In this configuration, the tissue culture media becomes analogous to blood exposure of the brain microvascular endothelium. This BBB organoid model demonstrates intact BBB functional integrity, tight junctions, trans-endothelial transport, and both responds to and produces inflammatory cytokines anticipated in many CNS disease conditions [20,21,22,23,24]. The 3D BBB organoids have also been used in a body-on–a-chip (BOC) platform, which revealed that exposing upstream liver cell organoids to a prodrug produced metabolites that were toxic to brain cells downstream in the BOC platform [25,26]. Thus, the 3D BBB organoids demonstrate excellent measurable transcellular and paracellular barrier functional integrity at baseline and decreased barrier integrity yielding increased permeability when challenged in disease models of inflammation, ischemia, and potentially toxic drug challenges.

In the present work, we aimed to demonstrate the feasibility of using our 3D BBB model to mimic interactions of immune cells at the BBB (e.g., neuroimmune interactions) under normal and inflammatory conditions. Inflammation was evoked by treating with proinflammatory cytokines (TNF-α and IFN-γ) and by oxygen–glucose deprivation–reoxygenation (OGD–R) as a model for ischemia–reperfusion. Ischemic and hemorrhagic stroke and associated reperfusion, whether spontaneous or medical, damages the BBB and increases immune cell entrance into brain tissue. Neuroinflammation triggered by reperfusion facilitates the continued production of cytokines and reactive oxygen species as well as the secretion of matrix metalloproteinases that contribute to further damaging the BBB, subsequently worsening the patient’s neurological outcome [27,28,29].

The disruption of the BBB was assessed by measuring permeability to fluorescent dextrans and quantifying the expression of tight junction proteins. The induction of hypoxia and production of reactive oxygen species in reperfusion as an indication of inflammation were assessed by fluorescent assays. Inflammation induced in the OGD-R model was also assessed by measuring TNF-α levels. Quantification of immune cell transmigration was assessed by confocal imaging of fluorescently labeled immune cells and flow cytometry. The combined data demonstrate the utility of this 3D BBB model for studying immune cell interactions in a physiologically relevant preclinical disease model. To our knowledge, this is the first report of an in vitro 3D spheroidal brain model to test immune cell infiltration through the BBB.

## 2. Materials and Methods

### 2.1. Cell and Organoid Culture

Protocols for cell culture and organoid composition are described in our previous publications, with minimal modifications [20,21]. Primary human brain microvascular endothelial cells (HBMVECs, Cell Systems, Kirkland, WA, USA) were grown in flasks coated with attachment factor using complete classic media supplemented with a culture boost and attachment factor, according to the manufacturer’s instructions. Primary human brain microvascular pericytes (HBVPs, ScienCell Research Laboratories, Carlsbad, CA, USA) were expanded via attachment factor-coated flasks and cultured in pericyte media supplemented with 2% FBS, pericyte growth supplement, and penicillin–streptomycin according to the manufacturer’s instructions. Human astrocytes (HA, ScienCell Research Laboratories, Carlsbad, CA, USA) were expanded in plates coated with 0.2 mg/mL Matrigel (Corning, Corning city, NY, USA) and cultured under normal growth conditions in astrocyte medium (ScienCell, Carlsbad, CA, USA) containing 2% FBS, astrocyte growth supplement, and penicillin–streptomycin. Human iPSC-derived oligodendrocyte progenitor cells (HO, Tempo Bioscience Inc., San Francisco, CA, USA) and human iPSC-derived microglia (HM, Tempo Bioscience Inc., San Francisco, CA, USA) were both expanded on flasks coated with 0.2 mg/mL Matrigel (Corning, Corning city, NY, USA) and cultured under normal growth conditions in DMEM/F-12 (Life Technologies, Carlsbad, CA, USA), with the addition of supplements specific for both, as described previously [20,21]. The ReNcell^®^ VM Human Neural Progenitor Cell Line (Sigma Aldrich, St. Louis, MO, USA) was cultured in flasks coated with 20 µg/mL laminin and ReNcell^®^ NSC maintenance media (Sigma Aldrich, St. Louis, MO, USA) containing recombinant human FGF2 (20 ng/mL, Axol Biosciences, Cambridge, UK) and recombinant human EGF (20 ng/mL, Axol Biosciences, Cambridge, UK). Organoid culture: all six cell types were harvested via Accutase (Sigma Aldrich, St. Louis, MO, USA). The organoids contained 30% HBMVECs, 15% HBVPs, 15% HA, 5% HM, 15% HO, and 20% HN, with approximately 2000 cells per organoid. Four cell types (HAs, ReNs, HOs, and HMs) were seeded at the aforementioned ratios in round-bottom ultra-low attachment plates (ULA, Corning, Corning city, NY, USA) in 50% astrocyte basal media without supplements (ScienCell Laboratories, Carlsbad, CA, USA) and 50% neural maintenance media (Axol Biosciences, Cambridge, UK). The media mixture was supplemented with 5% heat-inactivated FBS and 10 ng/μL collagen rat tail I (Corning, Corning city, NY, USA). The neuroglial organoid core was allowed to self-aggregate for 24 h. HBMVECs and HBVPs were harvested and added at ratios of 30% and 20%, respectively, to a mixture of media containing 50% astrocyte basal media without supplements and 50% Endothelial Cell Growth Medium-2 (EGM™-2, Lonza, Walkersville, MD, USA) supplemented with 2% FBS, 0.04% hydrocortisone, 0.01% R3-IGF-1, 0.1% ascorbic acid, 0.1% heparin, and 1 µL/mL GA-1000 (30 mg/mL gentamicin and 15 µg/mL amphotericin). Organoids were maintained in a mixture of 60% neural maintenance media, 20% astrocyte basal media, and 20% EGM-2 with supplements.

### 2.2. Treatment of Organoids with Inflammatory Cytokines

Organoids were used between day 10 and day 14. They were maintained in round-bottom ULA plates with media changes every other day, and daily after day 7 in vitro. Twenty-four hours before the experiments were conducted, the organoids were treated with 10 ng/mL of the inflammatory cytokines TNF-α (Peprotech, Cranbury, NJ, USA) or IFN-γ (Peprotech, Cranbury, NJ, USA). After 24 h, the organoids were collected and used in different downstream experiments.

### 2.3. Oxygen-Glucose Deprivation-Reoxygenation (OGD-R) Model

Organoids were incubated in a hypoxia chamber (Xvivo System G300C, BioSpherix, Redfield, NY, USA) at 37 °C, 0.1% O_2_ saturation, and 5% CO_2_ for 12 h in low-glucose DMEM (Gibco, ThermoFisher Scientific, Waltham, MA, USA) supplemented with 5% heat-inactivated FBS (OGD model). The normoxic controls were incubated in normal cell culture conditions with atmospheric O_2_ and 5% CO_2_ for the same 12 h. To mimic reperfusion, the organoids were transferred back to normal growth conditions of normal glucose, atmospheric O_2_ and 5% CO_2_ at 37 °C with normal daily exchanges of media and maintained for 24, 48, and 72 h. Normoxic controls were maintained for the same time periods.

### 2.4. Viability Assessment

The viability of the organoids was tested using the Molecular Probes Live–Dead cell imaging system (Calcein AM, green for live cells; ethidium homodimer-1, red for dead cells; Invitrogen, Carlsbad, CA, USA). Briefly, organoids were harvested after cytokine treatment for 24 h, after OGD for 12 h, or after 24, 48, and 72 h of reperfusion. Organoids were washed once with Dulbecco’s Phosphate-Buffered Saline (DPBS) and incubated in DPBS containing 2 µM Calcein AM and 4 µM ethidium homodimer-1 at room temperature for 10 min protected from light. Organoids were washed with prewarmed FluoroBrite™ DMEM (Thermo Fisher Scientific, Waltham, MA, USA) and examined in the same media with an Olympus Fluoview Fv10i (Olympus, Tokyo, Japan) laser scanning confocal microscope. At least 5 Z-stacks per condition were captured with confocal microscopy, and Z-stack projection images were created. The number of dead cells was counted using the ImageJ particle analysis plug-in (version 1.54r) and compared between distinct groups.

### 2.5. ATP Assay

ATP production was measured using the CellTiter-Glo 3D Reagent (Promega Life Sciences, Madison, WI, USA). Briefly, the organoids were transferred into opaque 96-well plates in 100 µL of media. Eight organoids were used for each experimental group (OGD for 12 h and reperfusion for 24, 48, and 72 h) and for matching normoxic controls. The experiment was repeated for 3 biological replicates (*n* = 3). The CellTiter-Glo 3D Reagent was thawed overnight and brought to room temperature before use. One hundred microliters of the reagent were added to 100 µL of media containing organoids. The organoids were incubated at room temperature for 30 min on an orbital shaker protected from light. Luminescence was measured via a GloMax^®^ Navigator plate reader (GM2000, Promega Life Sciences, Madison, WI, USA). Background luminescence from media without organoids was then subtracted from the samples.

### 2.6. Permeability Assessment

For permeability assessment, organoids were incubated with 70 kDa dextrans (Dextran, Oregon Green™ 488; 70,000 MW, Anionic, Lysine Fixable, Thermo Fisher Scientific, Waltham, MA, USA) at a concentration of 50 µg/mL for 30 min prior to the end of each experimental condition (24 h of cytokine treatment, 12 h of OGD or 24-, 48-, and 72 h reperfusion). Organoids were moved in media containing dextrans, without washing, to confocal dishes for examination via an Olympus Fluoview Fv10i laser scanning confocal microscope. At least 5 z-stacks were captured, and Z-projection images were created. The mean fluorescence intensity of green fluorescence inside and outside the organoids was measured using ImageJ (version 1.54r). The relative permeability of an organoid was calculated as the ratio of the inside/outside mean fluorescence intensity.

### 2.7. ROS and Hypoxic Immunofluorescence Staining

To validate the induction of hypoxia, organoids were incubated with Image-iT Green Hypoxia Reagent (5 µM, Invitrogen, Carlsbad, CA, USA) for one hour, and the media was exchanged with fresh media before incubation in the hypoxic chamber prior to the start of reperfusion. Normoxic control organoids were stained simultaneously. The hypoxia reagent becomes fluorescent when the oxygen level decreases. The fluorescent signals were significantly greater than those in the controls after 12 h of hypoxia. After termination of hypoxia or reperfusion, the organoids were washed once with DPBS, fixed with 4% paraformaldehyde, and imaged within 24 h using an Olympus Fluoview Fv10i laser scanning confocal microscope (Tokyo, Japan).

For detection of reactive oxygen species, the organoids were incubated for 30 min before termination of 12 h of hypoxia or reperfusion time points with CellROX™ Deep Red Reagent (Invitrogen, Carlsbad, CA, USA). Organoids were washed once with DPBS, fixed with 4% PFA and imaged using an Olympus Fluoview Fv10i (Olympus, Tokyo, Japan) laser scanning confocal microscope within 2 h of fixation. For both hypoxia and ROS staining, a minimum of 5 Z-stacks were captured, and Z-projection images were generated. An ImageJ analysis of the green (hypoxic) or red (reactive oxygen species (ROS)) fluorescence intensity inside the organoids was performed. Background fluorescence was calculated and subtracted from the mean fluorescence intensity inside the organoids.

### 2.8. TNF-α Measurement in the Supernatant of the OGD-Reperfusion Model

After 12 h of OGD and 24, 48, and 72 h of reperfusion, the media were collected. The media was centrifuged at 300× *g* for 10 min to remove cells and debris, aliquoted, and stored at −80 °C until use. TNF-α levels were measured using a TNF alpha Human ELISA Kit (Invitrogen, Carlsbad, CA, USA). Standard curves were created according to the manufacturer’s protocol. Each experiment was conducted for three biological replicates (*n* = 3). Each replicate represents media pooled from eight organoids.

### 2.9. Immunofluorescence Staining of BBB Markers After Cytokine Stimulation

BBB organoids were stained and fluorescently imaged for selective markers. Organoids were treated for 24 h with TNF-α or IFN-γ. The negative controls were left untreated. After 24 h, eight organoids from each group were washed three times with cold DPBS and fixed overnight at 4 °C with 4% paraformaldehyde (PFA). Once again, the organoids were washed three times with DPBS, permeabilized with 0.1% Tween-20 for 15 min, and then washed three times. The organoids were blocked for one hour at room temperature with Dako Protein Block Solution and washed three times. The samples were incubated overnight at 4 °C with primary antibodies in Dako antibody diluent at a ratio of 1:200. Anti-human occludin (Abcam, Waltham, MA, USA), anti-human claudin-12 (Thermo Fisher, Waltham, MA, USA), anti-human VCAM1 (Proteintech, Rosemont, IL, USA) and anti-human ICAM1 (Proteintech, Rosemont, IL, USA) were used. The organoids were washed three times with DPBS and incubated with secondary antibodies at a ratio of 1:1000 for 1 h at room temperature. The following secondary antibodies (Abcam, Waltham, MA, USA) were used: goat anti-rabbit IgG H&L (Alexa Fluor^®^ 488), preadsorbed goat anti-rabbit IgG H&L (Alexa Fluor^®^ 594), preadsorbed goat anti-mouse IgG H&L (Alexa Fluor^®^ 488), and preadsorbed goat anti-mouse IgG H&L (Alexa Fluor^®^ 594). At least three organoids from each group were examined via laser scanning confocal microscopy for each stain at 10× and 60× magnifications at the same laser intensity for comparison across treatments. Four to seven slices were captured and Z-stacked into a single image. Immunofluorescence staining was conducted for qualitative examination of the stain intensity and distribution. For quantitative evaluation, qPCR and protein assessment with ELISA were carried out for the same markers.

### 2.10. RNA Extraction and Quantitative Real-Time PCR for Cytokine Stimulation and OGD-R

To accurately quantify changes in the BBB markers’ expression under various exposures, qPCR was used to detect the expression of such markers. RNA extraction was carried out by collecting 32 organoids from each condition (24 h of cytokine treatment, 12 h of OGD, and 24, 48, and 72 h of reperfusion and matching controls) in Eppendorf tubes, and total RNA was extracted from the organoids via a Qiagen RNeasy Plus Mini Kit (Qiagen GmbH, Hilden Germany) following the manufacturer’s instructions. Organoids were homogenized with a tissue ruptor (Qiagen, GmbH, Hilden, Germany) to maximize the amount of RNA extracted. RNA quantity and quality were assessed using Nanodrop 2000/2000c spectrophotometer VI.0 (Thermo Fisher Scientific, Waltham, MA, USA). For reverse transcription, total RNA was reverse transcribed at a concentration of 2 µg in a 20 µL reaction volume using High-Capacity cDNA Reverse Transcription Kit with an RNase Inhibitor (Applied Biosystems; Thermo Fisher Scientific, Baltics UAB) according to the manufacturer’s instructions in a Veriti™ 96-well Thermal Cycler (Applied Biosystems, Thermo Fisher Scientific, Baltics UAB). Readymade TaqMan probes for the target genes occludin (Hs05465837_g1), claudin-12 (Hs00273258_s1), VCAM1 (Hs01003372_m1), and ICAM1 (Hs00164932_m1) and the reference gene HPRT1 (Hs02800695_m1) were purchased from Applied Biosystems. Real-time qPCR was performed on a QuantStudio 3 Real-Time PCR machine (Applied Biosystems, Waltham, MA, USA) with TaqMan™ Universal PCR Master Mix Taq (Thermo Fisher, Waltham, MA, USA) on Microamps^®^ Optical 96-Well Reaction Plates in a 20 µL reaction volume following the manufacturer’s instructions. One hundred micrograms of cDNA were loaded in each well, and the thermal cycling conditions were as follows: 10 min of polymerase activation at 95 °C, 40 PCR cycles with 15 s of denaturation, and 1 min of annealing and extension at 60 °C. The HPRT1 housekeeping gene was analyzed in parallel for each run to normalize the mRNA values. Each biological sample was run in duplicates or triplicates, and for each condition, three biological samples were obtained (*n* = 3). The delta–delta Ct method (2^−∆∆Ct^ method) was used to calculate the relative fold gene expression of the markers of interest in the cytokine-treated groups compared with the untreated ones.

### 2.11. Protein Extraction and ELISA Analysis of Tight Junctions and Adhesion Molecules

To detect the extent of correlation between changes at the molecular level and the cellular levels of select markers, we conducted ELISA protein quantification of select BBB markers. For protein extraction, two groups of organoids were treated with TNF-α and IFN-γ for 24 h, and one group was left untreated. Forty-eight organoids from each group were collected into tubes, washed with cold DPBS three times, and kept on ice. Cold RIPA lysis buffer supplemented with protease inhibitor cocktail (Promega, Madison, WI, USA) was used to lyse the organoids for 30 min on ice while vortexing every 10 min. The tubes were subsequently centrifuged for 15 min at 13,000× *g* at 4 °C, after which the supernatants were transferred to sterile tubes. The total protein concentration was measured with a Pierce BCA protein assay (Thermo Scientific, Rockford, IL, USA, 23227) following the manufacturer’s instructions. To quantify Occludin, Claudin-12, VCAM1, and ICAM1 protein expression under different conditions, the following ELISA kits were used: Human OCLN/Occludin ELISA (LifeSpan BioSciences Inc., Seattle, WA, USA), Human claudin-12 ELISA (AMSBIO, Cambridge, MA, USA), Human VCAM1 PicoKine ELISA (Boster, Pleasanton, CA, USA), and Human ICAM1 PicoKine ELISA (Boster, Pleasanton, CA, USA), respectively. A total of 100 µL of each lysate was added to the wells. At least three biological replicates were analyzed (*n* = 3). Each biological replicate is protein lysate collected from forty-eight organoids. The O.D. was measured by a plate reader at 450 nm, and target protein quantities in 100 µL lysate were calculated via a four-parameter logistic curve using free online software “(https://www.myassays.com/, accessed on 12 February 2025)”.

### 2.12. CD4^+^ T-Cell Isolation, Activation, and Transmigration After Cytokine Treatment or OGD-R

The study was reviewed and approved by the Institutional Review Board at Wake Forest University School of Medicine. Peripheral blood mononuclear cells (PBMCs) were isolated from venous blood samples withdrawn from healthy donors after informed consent was obtained via density gradient separation (Lymphoprep, Stem Cell Technologies, Vancouver, BC, Canada, 07801). Naïve CD4^+^ T cells were isolated from PBMCs using a negative selection kit, EasySep Human Naïve CD4^+^ T-cell isolation kit (19555, Stem Cell Technologies, Vancouver, BC, Canada), according to the manufacturer’s instructions.

Isolated naïve CD4^+^ T cells were cultured under normal growth conditions at a density of 1 × 10^6^ cells/mL in ImmunoCult™-XF T-Cell Expansion Medium (Stem Cell Technologies, Vancouver, BC, Canada). T cells were activated with ImmunoCult™ Human CD3/CD28 T-Cell Activator (Stem Cell Technologies, Vancouver, BC, Canada) at a concentration of 25 µL/mL for 4 days at 37 °C with the addition of recombinant human IL-2 (Peprotech, Cranbury, NJ, USA).

After three days of stimulation, the activated cells were washed twice with fresh media. Activated T cells were stained with PKH26 dye (Sigma Aldrich, St. Louis, MO, USA) according to the manufacturer’s instructions. The reaction was stopped with heat-inactivated FBS, and the cells were centrifuged at 1500 rpm for 5 min and washed twice with RPMI-1640 media. CD4^+^ T cells were counted and suspended in RPMI-1640 media supplemented with 10% FBS at a density of 1 × 10^6^ cells/mL. Activated CD4^+^ T cells were subsequently co-incubated with organoids after 24 h of cytokine treatment, 6 h before the end of the OGD 12 h time point, or 6 h before the end of the reperfusion 48 h-time point. After 6 h, the organoids from each group were collected in Eppendorf tubes. The samples were washed with DPBS three times to remove excess and loosely adherent T cells. Four organoids from each group were fixed in 4% paraformaldehyde for 1 h at 37 °C. The samples were washed twice, incubated with a DAPI counterstain for 10 min and examined under an Olympus Fluoview Fv10i laser scanning confocal microscope.

For quantification of transmigrated T cells by flow cytometry, 8 organoids from each group were washed three times with DPBS and then incubated with 1 mL of Accutase™ solution (Sigma Aldrich, St. Louis, MO, USA, A6964) at room temperature on a rotator for 30 min. Dissociated cells were filtered through a 70 µm cell strainer, washed twice with DPBS, and fixed for 30 min with 2% paraformaldehyde. Samples were acquired on a BD Accuri™ C6 flow cytometer (Becton Dickson & Company BD Biosciences, San Jose, CA, USA). Dissociated organoids without stained CD4^+^ T cells were used as autofluorescence controls. At least 10,000 events were acquired by flow cytometry from each group, and the percentage of cells stained with PKH26 was used to indicate the number of transmigrated CD4^+^ T cells in each group. The migration experiments were repeated three times (*n* = 3).

The same experiments were conducted with blocking antibodies. Before incubating with the stained activated CD4^+^ cells, the organoids were incubated with media containing 20 µg/mL blocking antibodies for one hour and then incubated with the activated T cells for 6 h. The functional-grade blocking antibodies used were human anti-VCAM1 (Bio-Rad, Hercules, CA, USA), human anti-ICAM1 (Bio-Rad, Hercules, CA, USA) and human anti-CD31 (eBioscience, San Diego, CA, USA).

### 2.13. Data Analysis and Statistics

All data in the manuscript were presented as mean and standard deviation. GraphPad Prism v10.4.1 software was used to conduct statistical tests and graph the results. Multiple replicates were acquired for each experiment as described above. A two-tailed Student’s *t*-test was used to calculate statistical significance when changes in the experimental group were compared to its own matching control, and those data are presented as a percent of control. Normality of the data was assessed with the Shapiro–Wilk test with no omission of outliers and with *p*-value of 0.05 to justify the use of *t*-test. One-way ANOVA was used in data sets that compared more than two end points between different treatment groups. The level of significance is 0.05, with symbols * for *p* < 0.05, ** for *p* < 0.01, *** for *p* < 0.001, and **** for *p* < 0.0001.

## 3. Results

### 3.1. Viability Assessment After Cytokine Treatment or Oxygen–Glucose Deprivation–Reoxygenation (OGD-R)

Inflammatory cytokines have been reported to induce apoptosis in cells. Therefore, viability was assessed to determine the effect of inflammation induced by cytokines and OGD-R model. After stimulation, organoids were stained for 10 min with Calcein for live cells and ethidium homodimer for dead cells after cytokine treatment for 24 h (Supplementary Figure S1A–C,A`–C`). The number of dead cells in each treatment group was plotted as a percent of dead cells in untreated controls. TNF-α and IFN-γ induced significant cell death in the organoids (*p* = 0.007 and 0.008 for TNF-α and IFN-γ, respectively; Supplementary Figure S1D).

**Figure 1:**
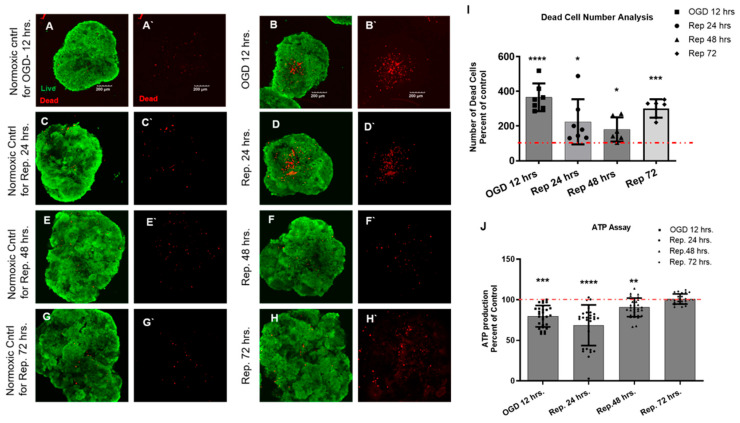
**Cell death and ATP levels in oxygen-glucose deprivation-reoxygenation (OGD-R) model**. (**A**–**H**) show organoids stained with Calcein (green-live) and ethidium bromide (red-dead) after experimental exposure to OGD (hypoxia) for 12 h and reperfusion (Rep.) at different time points. (**A`**–**H`**) show same organoids with red only channel (dead cell). Images were acquired by confocal microscopy and presented as Z-stack projection of serial slices. (**I**) Dead cells were quantified by image J particle analysis plug-in. OGD (hypoxia) for 12 h induced significant cell death, *p* < 0.0001. The number of dying cells decreased but was still significantly different from normoxic controls after 24, 48, 72 h with *p* values of 0.012, 0.029, 0.003, respectively. The number of dead cells in each experimental group was plotted as percent of the number of dead cells in its time-matched normoxic controls. Normoxic control values correspond to 100 (red dotted line). Two-tailed paired Student’s *t*-test was used to calculate statistical significance between each experimental condition and its normoxic control. (**J**) ATP production was measured using CellTiter-Glo™ 3D Cell viability bioluminescence assay. Change ATP production in each experimental group was plotted as a percent of bioluminescence in its time-matched normoxic control. Normoxic controls values correspond to 100 (red dotted line). Two-tailed paired Student’s *t*-test was used to calculate statistical significance between each experimental condition and its normoxic control. ATP production significantly decreased with OGD (hypoxia) for 12 h, *p* < 0.0002, and continued to be significantly decreased after reperfusion for 24 and 48 h, *p* < 0.0001 and 0.002, respectively. ATP production improved after 72 h of reperfusion. Results are represented as mean and standard deviation with individual points representing individual organoids from three biological replicates. Scale bar (**A**,**B**) = 200 μm, all images were captured at the same magnification. Level of significance is 0.05, with symbols * for *p* < 0.05, ** for *p* < 0.01, *** for *p* < 0.001, **** for *p* < 0.0001.

Organoid viability after OGD and reperfusion was determined by staining via the same method (Figure 1A–H,A`–H`). Cell death was significant after hypoxia for 12 h (*p* < 0.0001), and after reperfusion for 24 h, 48 h, and 72 h with *p* = 0.012, *p* = 0.029, and *p* = 0.0003 for each respectively (Figure 1I). Both cytokines and OGD-R challenge model inflammatory injury by inducing increased cell death compared to the normal controls which reflects the pathological conditions established in the organoids.

### 3.2. Decreased ATP Production in the OGD-R Model

To evaluate the effect of oxygen depletion on the production of ATP, brain organoids were incubated with CellTiter-Glo 3D reagent, and ATP bioluminescence was measured and compared among the OGD-R groups and matching normoxic controls. ATP production was significantly reduced after hypoxia (*p* = 0.0002) and continued to be significantly reduced throughout reperfusion at 24 and 48 h (*p* < 0.0001 and *p* = 0.002 respectively). ATP production improved with reperfusion at 72 h (Figure 1J) and was non-significant compared to matched normoxic control. The decrease in ATP production further reflects the induction of hypoxia inside the OGD-R models, which is a central part of pathophysiology.

### 3.3. Induction of Hypoxia and Reactive Oxygen Species Production in the OGD-R Model

**Figure 2:**
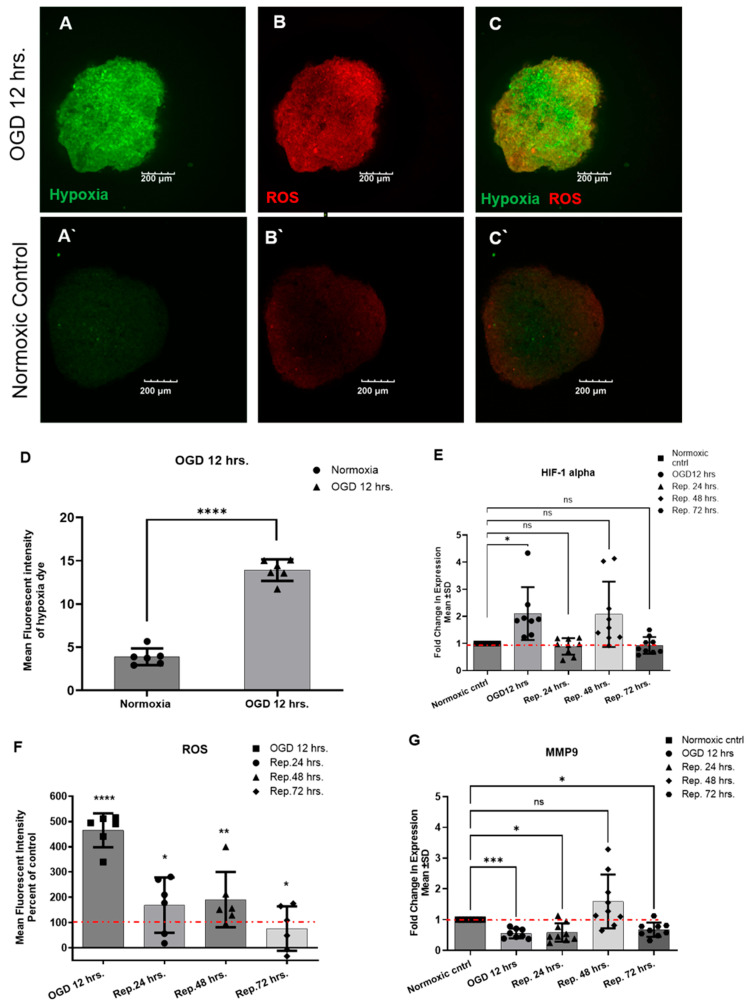
**Induction of hypoxia and ROS production in oxygen-glucose deprivation-reoxygenation (OGD-R) model**. (**A**–**C**) show confocal microscopy images for organoids stained with Hypoxia Click-it dye (green—(**A**)), CellROX™ Deep Red (red—(**B**)) and merge between two channels (**C**) after OGD (hypoxia exposure). (**A`**–**C`**) show same stains in normoxic control. Images are presented as Z-stack projection of serial slices and stain intensity was measured by ImageJ software. (**D**) The green mean fluorescent intensity (MFI) of hypoxic dye showed a significant increase over control normoxic organoids, *p* < 0.0001. Two-tailed paired Student’s *t*-test was used to calculate statistical significance. Individual points represent individual organoids from three biological replicates. (**E**) Gene expression analysis of HIF-1α mRNA showed significant fold increase after OGD compared to its own time-matched normoxic organoid controls, *p* = 0.046. One-way ANOVA with Dunnett’s post hoc test was used to calculate significance relative to normoxic controls. Individual points represent log fold change in expression from each technical replicate calculated, using ΔΔCT method, across three biological replicates. Baseline log fold expression of HIF-1α in all time-matched normoxic controls is normalized to value of one and represented by red dotted line. (**F**) CellRox MFI for ROS detection was significantly increased after OGD exposure for 12 h, *p* < 0.0001. ROS decreased with reperfusion (Rep.) but remained significantly elevated above baseline, *p* = 00.01, *p* = 0.001, and *p* = 0.02 for reperfusion for 24, 48, and 72 h respectively. MFI in experimental groups are presented as percent in normoxic controls. MFI of control group is normalized to the value of one hundred and represented by red dotted line. Two-tailed paired Student’s *t*-test was used to calculate significance between MFI in each experimental group and its matched normoxic control. Individual points represent individual organoids from three biological replicates. (**G**) Matrix Metalloproteinase-9 RNA expression was measured using TaqMan qPCR assay. MMP-9 expression was decreased in all experimental groups except reperfusion 48 h with *p* values of 0.0003, 0.016 and 0.025 for OGD, reperfusion 24 h and 72 h respectively. The expression after 48 h reperfusion appeared variably elevated; however, it was not statistically significant. One-way ANOVA with Dunnett’s test was used to calculate statistical significance. Individual points represent log fold change in mRNA expression from each technical replicate calculated, using ΔΔCT method, across three biological replicates. Baseline log fold expression of MMP-9 in all time-matched normoxic controls is normalized to value of one and represented by red dotted line. All graphs are represented as mean and SD. Scale bar (**A**) = 200 μm and all images were captured at same magnification. Level of significance is 0.05, with symbols * for *p* < 0.05, ** for *p* < 0.01, *** for *p* < 0.001, **** for *p* < 0.0001, and ns for not significant (>0.05).

Ischemia is associated with a deficiency in the oxygen supply and a shift to anaerobic respiration inside cells, which leads to decreased production of ATP. To establish the induction of hypoxia and ROS, the organoids were stained with Image-iT Green Hypoxia Reagent and CellROX deep red for ROS, respectively (Figure 2A–C for the OGD group and Figure 2A`–C` for the control group). Compared with that in normoxic organoids, the intensity of green fluorescence in hypoxic organoids was significantly greater (*p* < 0.0001, Figure 2D). To further confirm the induction of hypoxia at a molecular level in the organoids under oxygen deprivation and to establish the model, qPCR analysis was conducted for hypoxia-inducing factor 1α (HIF-1α). Compared with that in normoxic organoids, the expression of HIF-1 increased in hypoxic organoids (*p* = 0.046). The levels of HIF-1α decreased with reperfusion, with some elevation at 48 h of reperfusion that was not significant (Figure 2E).

The production of reactive oxygen species (ROS) is a pathophysiological outcome of ischemia and reperfusion injury. ROS are produced by microglia, astrocytes, and infiltrating immune cells [30,31]. To demonstrate whether ROS production increased in our model, the intensity of CellROX staining was measured in the organoids after 12 h of hypoxia and after reperfusion for 24, 48, and 72 h. ROS staining was significantly increased after hypoxia (*p* < 0.0001). The staining decreased with reperfusion but continued to be greater than that in normoxic controls throughout reperfusion (*p* = 0.01, 0.001, and 0.02 for reperfusion at 24, 48, and 72 h, respectively (Figure 2F)). Confocal images for all groups are shown in Supplementary Figure S2 for comparison. The induction of hypoxia was confirmed by staining and qPCR, and increased production of ROS was detected by confocal microscopy. These findings establish the generation of ischemia–reperfusion OGD-R model.

### 3.4. Production of Matrix Metalloproteinase 9 (MMP-9) in the OGD-R Model

The production of matrix metalloproteinases during ischemia and reperfusion has been frequently reported as a clinical biomarker for reperfusion injury [32]. To determine whether MMP-9 expressed in response to our OGD-R model we conducted qPCR analysis via TaqMan qPCR assay. MMP-9 expression was found to be significantly decreased with 12 h hypoxia (*p* = 0.0003), 24 h reperfusion (*p* = 0.016), and with 72 h reperfusion (*p* = 0.025) (Figure 2G). However, 48 h reperfusion showed a trend toward increased MMP-9 with greater variability in that data. This increase in MMP-9 signals the initiation of molecular mechanisms that might contribute to BBB disruption along with other factors.

### 3.5. Permeability of 70 kDa Dextrans with Cytokine Treatment or OGD-R

To investigate the effects of the proinflammatory cytokines TNF-α and IFN-γ on the permeability of our BBB model, 70 kDa FITC-dextrans were incubated for 30 min with both untreated organoids and 24 h treated organoids (Supplementary Figure S3A–C). Relative permeability was calculated as the ratio between the mean fluorescence inside the organoid and the mean fluorescence outside the organoid. The results are presented as a percent of relative permeability in control untreated organoids (Supplementary Figure S3D). In both the TNF-α- and IFN-γ-treated groups, there was a significant increase in permeability (*p* = < 0.0001 and *p* = 0.002 for TNFα- and IFN-γ, respectively).

**Figure 3:**
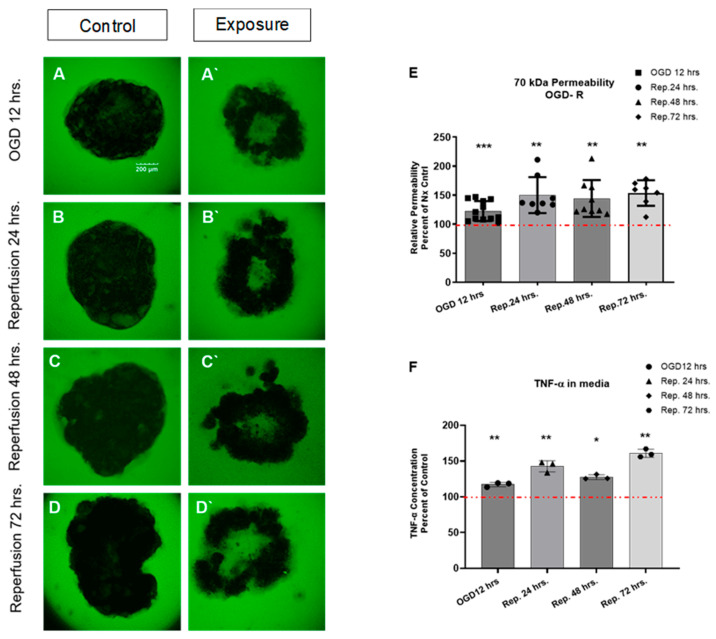
**70 kDa FITC-dextran permeability assay and TNF-α detection in oxygen-glucose deprivation-reoxygenation (OGD-R) model**. (**A**–**D**) show permeability of 70 kDa dextrans in normoxic control organoids. (**A`**–**D`**) show permeability in experimental organoids after OGD-R exposure. Images are presented as Z-stack projection of serial slices and stain intensity was measured by ImageJ software. (**E**) Permeability increased above baseline with OGD, *p* = 0.0007, with *p* = 0.003, 0.006 and 0.001 for reperfusion (Rep.) 24, 48, and 72 h respectively. Relative permeability of each experimental group is displayed as a percent of relative permeability in its own time-matched normoxic control. Relative permeability of normoxic controls were normalized to the value of hundred and presented by red dotted line. Two-tailed paired Student’s *t*-test was used to calculate statistical significance between experimental groups and their matched normoxic control. Individual points represent individual organoids from three biological replicates. (**F**) TNF-α concentrations in the supernatant were increased significantly in all experimental exposures with OGD, *p* = 0.008 and reperfusion for 24, 48, and 72 h having *p* values of 0.006, 0.015 and 0.003, respectively. TNF-α concentration in each experimental group were plotted as percent of TNF-α concentration in its time-matched normoxic control, which was normalized to the value of one hundred and represented by red dotted line. Two-tailed paired Student’s *t*-test was used to calculate statistical significance between TNF-α concentration in each experimental condition and its matched normoxic control. Each individual point represents the average of three technical replicates from each of three biological samples. Scale bar (**A**) = 200 μm with all images captured at the same magnification. Level of significance is 0.05, with symbols * for *p* < 0.05, ** for *p* < 0.01, *** for *p* < 0.001.

After acute ischemic stroke, BBB permeability increases due to the disruption of tight junctions resulting from the production of ROS and matrix metalloproteinases. A significant increase in permeability was observed after hypoxia (*p* = 0.0007). The permeability continued to increase beyond baseline in hypoxic organoids throughout reperfusion for 24, 48, and 72 h (*p* = 0.003, *p* = 0.006, and *p* = 0.001, respectively) (Figure 3A–D,A`–D`,E). Increased BBB permeability in our model recapitulates a major finding in many neurological disorders indicating the breakdown of tight junctions at the endothelial cell level.

### 3.6. TNF-α Secretion After OGD-R Injury

To further validate elements of inflammation in hypoxia-reperfusion injury, TNF-α protein secretion was measured with ELISA in the OGD-R model at various time points as follows: after 12 h of hypoxia and after 24, 48, and 72 h of reperfusion. These measurements were compared with those in normoxic control organoids. TNF-α levels increased significantly after hypoxia (*p* = 0.008) and remained significantly elevated with reperfusion with *p* = 0.006, 0.02, and 0.003 for reperfusion 24, 48, and 72 h, respectively (Figure 3F). TNF-α is a proinflammatory cytokine that, among other cytokines, adversely affects the BBB by increasing permeability, disrupting tight junctions, and promoting the adhesion and immigration of immune cells. The establishment of increased TNF-α secretion in OGD-R exposure provides evidence of the inflammatory nature of the disease.

### 3.7. Downregulation of Tight Junction mRNA and Protein Expression

**Figure 4:**
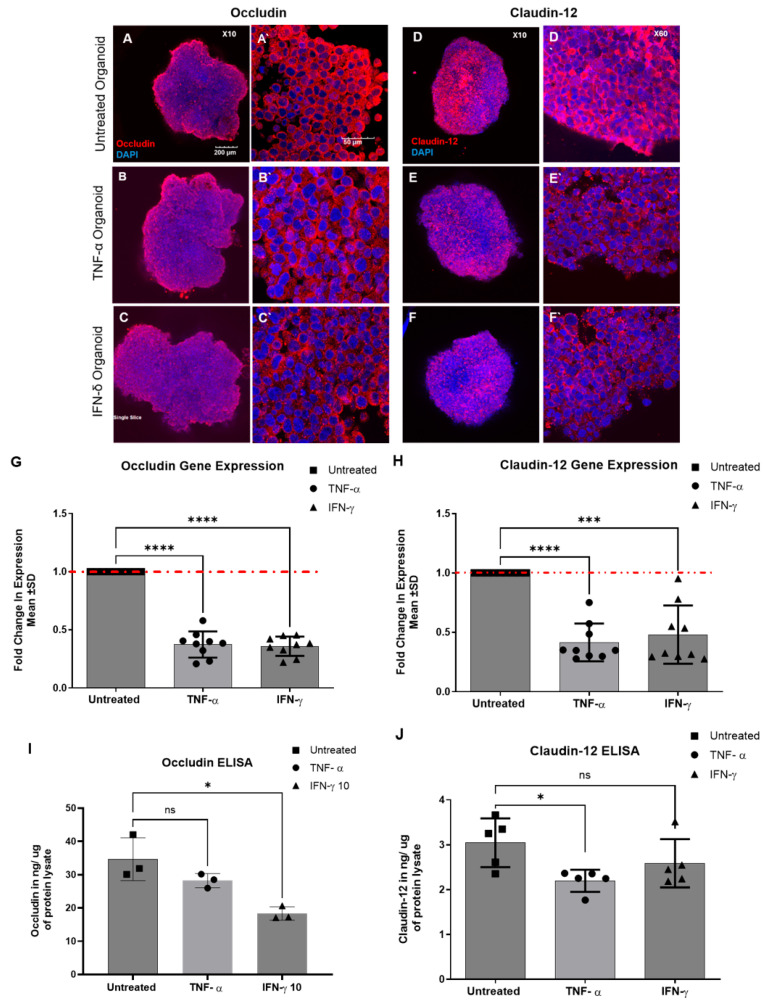
**Immunofluorescent staining of tight junction proteins and quantification with qPCR and ELISA**. (**A**–**C**) show organoids staining for occludin at 10× magnification and (**A`**–**C`**) show occludin stain at 60× magnification under control and cytokine treatment conditions. (**D**–**F**) show organoids staining for claudin-12 at 10× magnification and (**D`**–**F`**) show occludin stain at 60× magnification under control and cytokine treatment conditions. Organoids were imaged by confocal laser scanning microscopy and images represented as Z-stack projections of serial slices. (**G**,**H**) show mRNA expression analysis after cytokine treatment. (**G**) Graph shows fold change in the expression of occludin in the untreated organoids versus the TNF-α and IFN-γ treated organoids. Occludin mRNA expression significantly decreased with TNF-α, *p* < 0.0001, and IFN-γ, *p* < 0.0001. (**H**) Claudin-12 mRNA expression showed a significant decrease with TNF-α, *p* < 0.0001, and IFN-γ, *p* = 0.0004. One-way ANOVA with Dunnett’s post hoc test was used to calculate significance relative to untreated organoids. Individual points represent log fold change in expression from each technical replicate calculated across three biological replicates (*n* = 3). Baseline log fold expression of occludin and claudin-12 in untreated controls are normalized to value of one and represented by red dotted line. (**I**,**J**) ELISA for Occludin and Claudin-12 protein quantification. (**I**) Graph shows occludin protein expression significantly decreased with IFN-γ treatment, *p* = 0.037. (**J**) Graph shows Claudin-12 protein expression decreased significantly with TNF-α, *p* = 0.03. One-way ANOVA with Dunnett’s post hoc test was used to calculate significance relative to untreated organoids. Individual points represent averaged technical replicates from three biological replicates for occludin and 5 biological replicates for claudin-12. All graphs are presented as mean ± SD. Scale bars are 200 μm in (**A**) and 50 μm in (**A`**). Level of significance is 0.05, with symbols * for *p* < 0.05, *** for *p* < 0.001, **** for *p* < 0.0001, and ns for not significant (>0.05).

Tight junction proteins are the major contributors to the formation of tight paracellular spaces at the BBB. To probe changes in select tight junctions in our model in response to exposures, the effects of immunomodulators (TNF-α and IFN-γ) on the tight junctions of the BBB, occludin and claudin-12, were measured. Occludin expression is related to the permeability and integrity of the BBB. The response of occludin to different stimuli has been extensively investigated [33]. Immunofluorescence images confirm the expression of occludin in both control and TNF-α and IFN-γ treated organoids (Figure 4A–C for 10× magnification and Figure 4A`–C` for 60× magnification) with no significant differences in the localization or distribution in the cells. Although the expression of claudin-12 has been shown in the BBB, it is not suggested to be directly related to permeability in the BBB, although its full role remains to be elucidated [34]. In our model, claudin-12 expression was confirmed in both untreated and cytokine-treated organoids (Figure 4D–F for 10× magnification and Figure 4D`–F` for 60× magnification). For accurate quantification of expression, both RNA analysis and ELISA were conducted.

A significant decrease in occludin mRNA expression was shown using a TaqMan mRNA expression assay with both TNF-α treatment (*p* < 0.0001) and IFN-γ treatment (*p* < 0.0001) (Figure 4G). Claudin-12 mRNA expression significantly decreased after both TNF-α treatment (*p* < 0.0001) and IFN-γ treatment (*p* = 0.0004) exposure (Figure 4H). Protein quantification by ELISA revealed a significant decrease in occludin protein level with IFN-γ treatment (*p* = 0.0037). There was a slight decrease after TNF-α treatment, which was not found to be significant. Higher concentrations of TNF-α or different durations of TNF-α exposure may be required to alter occludin expression (Figure 4I). Protein quantification for claudin-12 revealed a significant decrease in expression with TNF-α treatment (*p* = 0.03). There was a decrease in claudin-12 protein expression with IFN-γ treatment, but it was not found to be statistically significant (Figure 4J).

**Figure 5:**
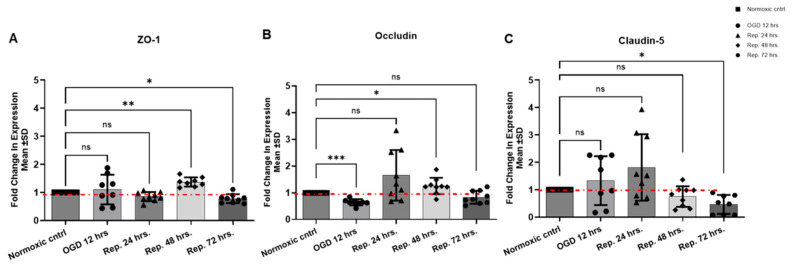
**mRNA expression of tight junction proteins after hypoxia and reperfusion**. RNA was extracted from the organoids after experimental exposures and TaqMan qPCR assay was conducted for tight junction gene expression. (**A**) Graph shows fold change in the expression of ZO-1 in the normoxic organoids versus the OGD-R organoids. ZO-1 expression significantly increased at reperfusion (Rep.) 48 h with *p* = 0.002 and decreased after reperfusion for 72 h, *p* = 0.026. (**B**) Occludin expression decreased after OGD exposure *p* = 0.0002 and increased at reperfusion 48 h, *p* = 0.03. (**C**) Claudin-5 expression significantly decreased after reperfusion for 72 h, *p* = 0.026. One-way ANOVA with Dunnett’s post hoc test was used to calculate significance relative to untreated organoids. Individual points represent log fold change in expression from each technical replicate, calculated using ΔΔCT method, across three biological replicates. Baseline log fold expression of each marker in untreated controls is normalized to value of one and represented by red dotted line. All graphs are mean ± SD. Level of significance is 0.05, with symbols * for *p* < 0.05, ** for *p* < 0.01, *** for *p* < 0.001, and ns for not significant (>0.05).

To quantify the gene expression of tight junction proteins in OGD-R, qPCR analysis for select tight junctions of known significance and clinical relevance was conducted (ZO-1, occludin and claudin-5). Claudin-12 was not included due to the limited evidence available regarding its regulation in this specific OGD-R model. qPCR analysis of tight junction expressions under OGD-R conditions revealed no significant reduction in ZO-1 after 12 h hypoxia and reperfusion for 24 h compared to normoxic controls. There was a significant increase after 48 h of reperfusion *p* = 0.002, and a significant decrease after 72 h of reperfusion *p* = 0.026 (Figure 5A). Occludin expression was decreased after 12 h hypoxia *p* = 0.0002. Similar to the ZO-1 trend, it was increased after 48 h of reperfusion *p*= 0.033 (Figure 5B). Claudin-5 expression showed only significant reduction after reperfusion for 72 h *p* = 0.026 (Figure 5C). These assays reflect changes in tight junctions in response to inflammation from direct cytokine exposure or OGD-R.

### 3.8. Upregulation of Cell Adhesion Molecule mRNA and Protein Expression

**Figure 6:**
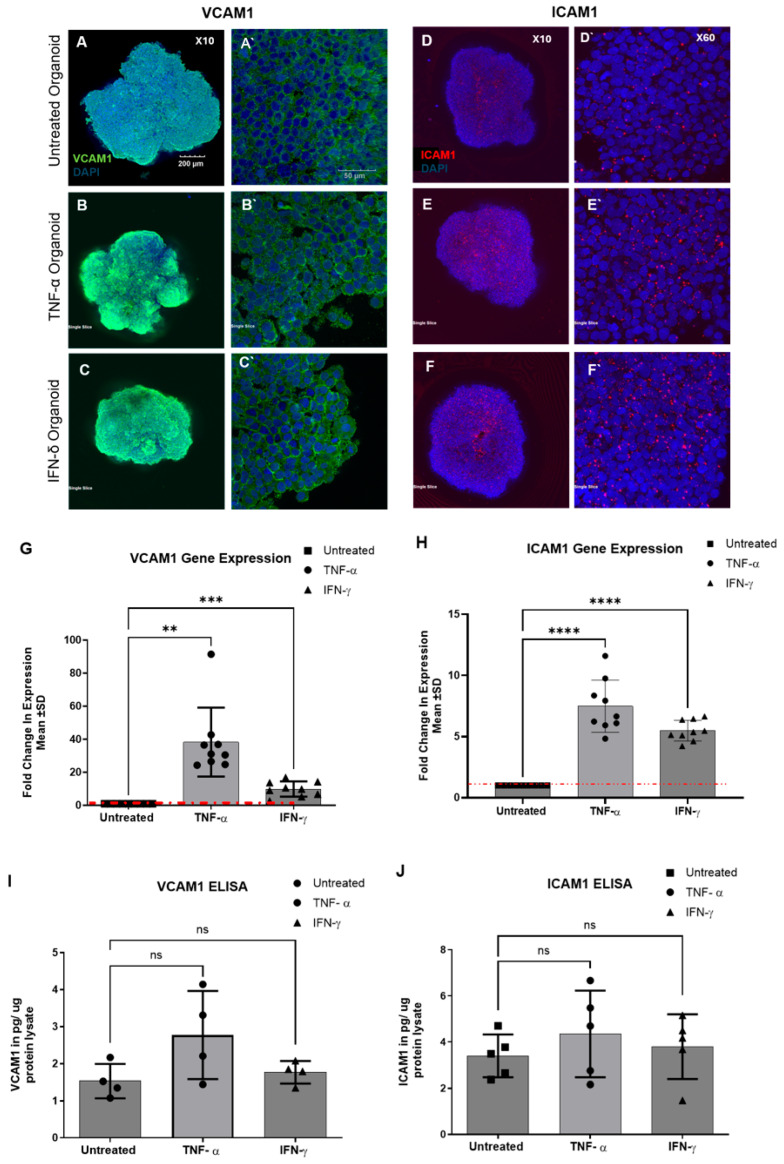
**Immunofluorescent staining of cell adhesion molecules and quantification with qPCR and ELISA**. (**A**–**C**) show organoids staining for VCAM1 at 10× magnification and (**A`**–**C`**) show VCAM1 stain at 60× magnification under control and cytokine treatment conditions. (**D**–**F**) show organoids staining for ICAM1 at 10× magnification and (**D`**–**F`**) show ICAM1 stain at 60× magnification under control and cytokine treatment conditions. Organoids were imaged by confocal laser scanning microscopy and images represented as Z-stack projections of serial slices. (**G**,**H**) RNA expression analysis after cytokine treatment. (**G**) Graph shows fold change in the expression of VCAM1 in the untreated organoids versus the TNF-α and IFN-γ treated organoids. VCAM1 expression significantly increased with TNF-α, *p* = 0.0012 and IFN-γ, *p* = 0.0007. (**H**) ICAM1 expression showed significant increase with TNF-α, *p* < 0.0001, and IFN-γ, *p* < 0.0001. Individual points represent log fold change in expression from each technical replicate across three biological replicates. Baseline log fold expression of VCAM1 and ICAM1 in untreated controls are normalized to value of one and represented by red dotted line. (**I**,**J**) ELISA for VCAM1 and ICAM1 protein quantification shows a trend upregulation of both proteins with both cytokines, but none was statistically significant. Individual points represent averaged technical replicates from four biological replicates for VCAM1 and 5 biological replicates for ICAM1. One-way ANOVA with Dunnett’s post hoc test was used to calculate significance relative to untreated organoids. All graphs are presented as mean ± SD. Scale bars are 200 μm in (**A**) and 50 μm in (**A`**). Level of significance is 0.05, with symbols ** for *p* < 0.01, *** for *p* < 0.001, **** for *p* < 0.0001, and ns for not significant (>0.05).

In context of inflammatory disorders at the BBB, cell adhesion molecules play very important roles in the regulation of immune cell interactions and immigration across the BBB. Hence, it was crucial to probe changes in cell adhesion molecules in response to the different exposures tested. To visualize the expression of cell adhesion molecules, IF staining of both untreated and cytokine-treated brain organoids was examined under confocal laser scanning microscopy with antibodies against VCAM1 (Figure 6A–C for 10× magnification and Figure 6A`–C` for 60× magnification) and ICAM1 (Figure 6D–F for 10× magnification and Figure 6D`–F` for 60× magnification). The images reflect no changes in the distribution or localization around the cells. For accurate quantification, qPCR analysis of RNA expression and ELISA were conducted for both markers under control untreated conditions and cytokine-treated conditions.

After treatment with inflammatory cytokines, both adhesion molecules showed significant upregulation in their mRNA expression, with VCAM1 being upregulated to a greater extent compared to ICAM1; VCAM1 *p* = 0.001 and 0.0007 for TNF-α and IFN-γ treatment, respectively (Figure 6G). ICAM1 was also significantly upregulated by both cytokines (*p* < 0.0001 and *p* = 0.0001 for TNF-α and IFN-γ treatment, respectively, Figure 6H). ELISA analysis of the protein expression of both adhesion molecules detected some increase but was not statistically significant (Figure 6I,J).

**Figure 7:**
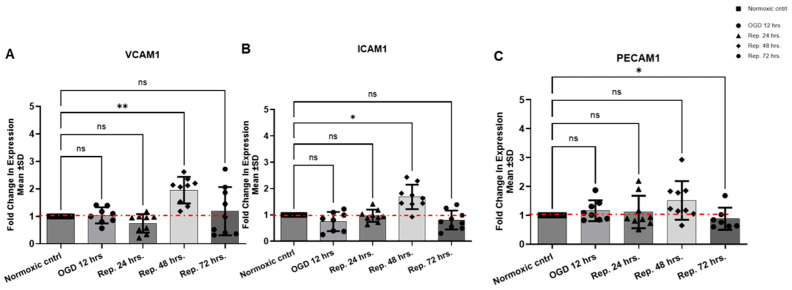
**mRNA expression of cell adhesion molecules proteins after hypoxia and reperfusion**. RNA was extracted from the organoids after experimental exposure and TaqMan qPCR assay was conducted for cell adhesion molecules. (**A**) Graph shows VCAM1 expression significantly increased at 48 h reperfusion (Rep.) in the OGD-R organoids verses the normoxic organoids, *p* = 0.004. (**B**) ICAM1 expression increased at reperfusion for 48 h, *p* = 0.013. (**C**) PECAM1 expression significantly decreased after reperfusion for 72 h, *p* = 0.038. One-way ANOVA with Dunnett’s post hoc test was used to calculate significance relative to untreated organoids. Individual points represent log fold change in expression from each technical replicate across three biological replicates. Baseline log fold expression of each marker in untreated controls are normalized to value of one and represented by red dotted line. All graphs are represented as mean ± SD. Level of significance is 0.05, with symbols * for *p* < 0.05, ** for *p* < 0.01, and ns for not significant (>0.05).

Reperfusion injury has been reported to increase the expression of cell adhesion molecules either directly or through the initiation of neuroinflammation via cytokine release and ROS production. The mRNA expression of both cell adhesion molecules did not significantly change after OGD for 12 h, but the expression of VCAM1 (*p* = 0.004) and ICAM1 (*p* = 0.013) significantly increased after 48 h of reperfusion (Figure 7A,B). PECAM1 analysis was added to OGD-R expression due to its clinical relevance in ischemia–reperfusion injury. The expression of PECAM1 did not significantly change except after 72 h perfusion where there was a significant decrease in expression, *p* = 0.038 (Figure 7C). These results demonstrate the capability of detecting changes in cell adhesion molecule expression that generally increased with inflammation and during reperfusion after hypoxia.

### 3.9. Increased Transmigration of Activated CD4^+^ T-Cells Across the BBB with Pro-Inflammatory Cytokines and Reduction with Anti-Cell Adhesion Molecule Antibodies

As inflammatory disorders affecting the brain alter BBB surface in a way that allows more immune cells to get into the brain than in healthy conditions, it was important to recapitulate those changes in our model after induction of inflammation. To probe the effects of upregulated cell adhesion molecules on T-cell transmigration, naïve CD4^+^ T cells were isolated from the peripheral blood of healthy donors as described in Section 2. Activated CD4^+^ T cells were stained with PKH26 dye after activation and incubated for 6 h with the cytokine-treated and untreated brain organoids.

**Figure 8:**
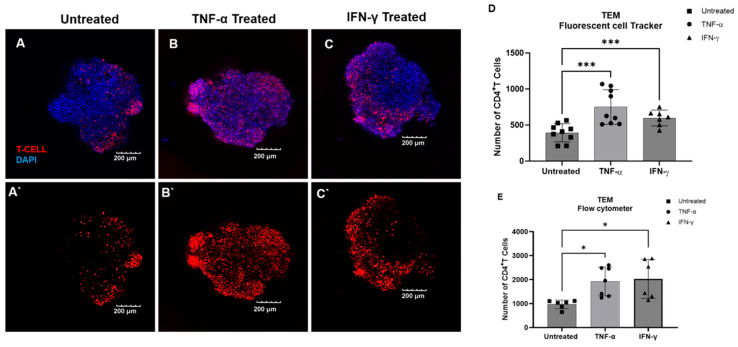
**Immunofluorescent images and flow cytometry of activated and transmigrated CD4^+^ T cells in cytokine-treated organoids**. CD4^+^ T-cells were activated and stained with red PKH26 and allowed to migrate into the untreated and cytokine treated organoids. (**A**–**C**) Merged images for dapi (blue) and immune cells (red). (**A`**–**C`**) show immune cells in red only channel. Organoids were imaged by confocal laser scanning microscopy and images represented as Z-stack projections of serial slices. (**D**) Transendothelial migration (TEM) of activated CD4^+^ T cells across the BBB was quantified by ImageJ analysis, and the number of transmigrated CD4^+^ T cells is increased after TNF-α and IFN-γ treatment of the organoid, *p* = 0.0002 and 0.0005, respectively. Individual points represent individual organoids from biological replicates. (**E**) Flow cytometry analysis of the number of CD4^+^ T cells in organoids confirmed changes detected by immunofluorescent images. TEM of CD4^+^ T cells showed significant increases with both TNF-α, *p* = 0.02 and IFN-γ, *p* = 0.012. One-way ANOVA with Dunnett’s test was used to calculate statistical significance. Individual points represent individual flow cytometry counts from 8 pooled organoids. Data are driven from six biological replicates. Graphs are presented as mean ± SD. Scale bar 200. Level of significance is 0.05, with symbols * for *p* < 0.05, *** for *p* < 0.001.

Confocal imaging of the organoids was conducted to visualize the T cells at a depth of 100–150 µm below the surface of the organoids, and Z-projection of the slices was performed (Figure 8A–C,A`–C`). The number of transmigrated T cells was analyzed using the ImageJ particle analysis plugin, and one-way ANOVA was performed to compare the control and cytokine-treated organoids. There was a significant increase in the number of transmigrated T cells with TNF-α treatment (*p* = 0.0002), and with IFN-γ treatment (*p* = 0.0005, Figure 8D). For better quantification and confirmation of the number of transmigrating cells, the organoids (eight per treatment group) were dissociated, and PKH-stained T cells were quantified by flow cytometry. There was a significant increase in the number of transmigrated cells after exposure to TNF-α (*p* = 0.02) or IFN-γ (*p* = 0.012) (Figure 8E). Flow cytometry blots for all the treatments are shown in Supplementary Figure S4.

To block T-cell transmigration, untreated and cytokine-treated organoids were incubated with anti-cell adhesion molecule antibodies, anti-VCAM1, anti-ICAM1, or anti-PECAM1 antibodies for 1 h at a concentration of 20 µg/mL before activated T cells were added. Excessive unbound antibodies were removed by washing the organoids with fresh media before the addition of T cells.

Using confocal microscopy, in untreated organoids, the number of PKH26-positive cells were quantified across groups. The decrease in T-cell immigration was significant with anti-VCAM1 (*p* = 0.037), anti-ICAM1 (*p* = 0.014) and anti-PECAM1 (*p* < 0.0001), respectively. Dissociation of the organoids and flow cytometry were conducted as previously described, and the number of PKH26-stained cells among the 10,000 events was compared across groups. In untreated organoids, all three anti-cell adhesion molecule antibodies decreased the transmigration of T cells to a variable extents, anti-VCAM1 (*p* = 0.005), and anti-ICAM1 (*p* = 0.005), and anti-PECAM1 (*p* < 0.0001), respectively (Supplementary Figure S5).

For the TNF-α treated organoids, confocal analysis revealed that the number of T cells significantly decreased with the three anti-cell adhesion molecule antibodies, namely, anti-VCAM1 (*p* = 0.007), anti-ICAM1 (*p* = 0.0005), and anti-PECAM (*p* = 0.013), respectively. Results were confirmed with flow cytometry for the three antibodies. There were a significant reduction in the number of PKH26-stained cells, with flow cytometry for anti-VCAM1 (*p* = 0.012), anti-ICAM1 (*p* = 0.01) and anti-PECAM1 (*p* = 0.002) (Supplementary Figure S6).

**Figure 9:**
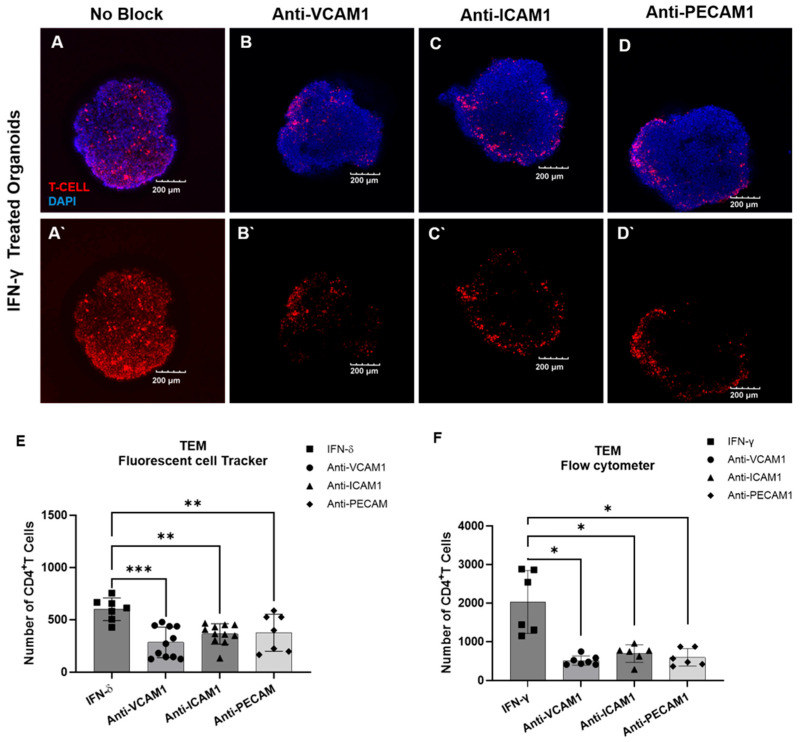
**Anti-cell adhesion molecule antibodies decreased the transmigration of activated CD4^+^ T cells into IFN-γ treated BBB organoids**. Activated T-cells were stained with red PKH26 cell tracker and incubated with IFN-γ treated organoids. (**A**–**D**) Merged images for dapi (blue) and immune cells (red). (**A`**–**D`**) Images show immune cells in red only channel. Organoids were imaged by confocal laser scanning microscopy and images represented as Z-stack projections of serial slices. (**E**) Transendothelial migration (TEM) of activated CD4^+^ T cells across the BBB, quantified by ImageJ analysis, was significantly lower after treatment with anti-VCAM1, anti-ICAM1 and anti-PECAM1 antibodies, *p* = 0.0007, 0.002 and 0.004, respectively. One-way ANOVA with Dunnett’s test was used to calculate statistical significance. Individual points represent individual organoids from three biological replicates. (**F**) Flow cytometry analysis of the number of CD4^+^ T cells in organoids was performed to confirm changes detected by confocal images. A significant decrease was detected with the three anti-cell adhesion antibodies with *p* values of 0.01, 0.03 and 0.013 for anti-VCAM1, anti-ICAM1 and anti-PECAM1, respectively. One-way ANOVA with Dunnett’s test was used to calculate statistical significance. Points represent individual flow cytometry readings from 8 pooled organoids from. Data driven from six biological replicates. Graphs are presented as mean ± SD. Scale bar (**A**) 200 μm. Level of significance is 0.05, with symbols * for *p* < 0.05, ** for *p* < 0.01, *** for *p* < 0.001.

For the IFN-γ treated organoids, all three antibodies significantly decreased transmigration, as determined via confocal scanning with ImageJ analysis (*p* = 0.0007 for anti-VCAM1, *p* < 0.002 for anti-ICAM1 and *p* = 0.004 for anti-PECAM1; Figure 9A–D,A`–D`,E). Using flow cytometry, similar reductions were observed for anti-VCAM1 (*p* = 0.011), anti-ICAM1 (*p* = 0.03), and anti-PECAM1 (*p* = 0.013), respectively (Figure 9F). Overall, the previous results reflect changes in the magnitude of immune cells infiltrating through the BBB into the organoid core as a function of leukocyte adhering to the BBB organoid endothelial surface.

### 3.10. Decreased Transmigration of Activated CD4^+^ T Cells Across the BBB Organoids with OGD (Hypoxia) with No Further Reduction with Anti-Cell Adhesion Molecule Antibody (Anti-ICAM1 Antibody)

**Figure 10:**
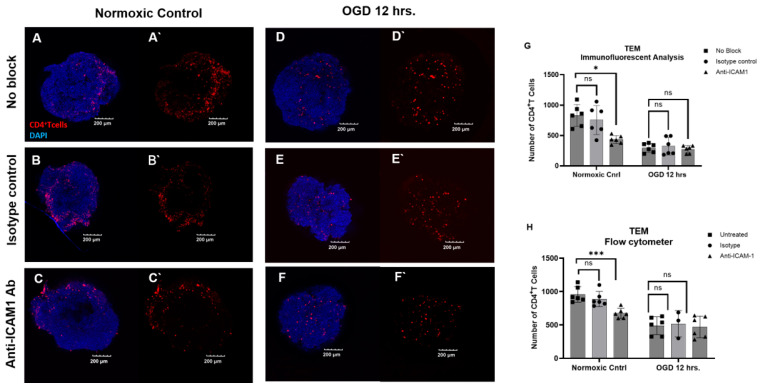
**Hypoxia (oxygen glucose deprivation-OGD) and anti-ICAM1 antibodies impacted CD4^+^ T cell transmigration into BBB organoids**. Activated T-cells were stained with red PKH26 cell tracker and incubated with the normoxic and hypoxic organoids for 6 h. For blocking assay, the anti-ICAM1 antibody was incubated with the organoids at 1 h prior to the transmigration experiment. (**A**–**C**) Normoxic organoids merged images for dapi (blue) and immune cells (red), and (**A`**–**C`**) images for red channel only. (**D**–**F**) Merged images for OGD (hypoxia) organoids, and (**D`**–**F`**) images for red channel only. Organoids were imaged by confocal laser scanning microscopy and images represented as Z-stack projections of serial slices. (**G**) Transendothelial migration (TEM) of activated CD4^+^ T cells across the BBB was significantly lower after incubation in OGD for 12 h compared to transmigration in normoxic control, *p* = 0.0009 (two-tailed paired Student’s *t*-test). Transmigration of activated T-cells was significantly reduced with anti-ICAM1 antibodies in normoxic organoids but not in hypoxic organoids, *p* = 0.013, one-way ANOVA with Dunnett’s test. Transmigration of activated CD4^+^ T-cells was not affected by incubating with isotypes control for anti-ICAM1 antibodies, which confirms that the reduction in transmigration is due to the specific binding to ICAM1. Individual graph points represent individual organoids from three biological replicates. (**H**) Flow cytometry analysis of the number of CD4^+^ T cells in organoids was performed to confirm changes detected by immunofluorescent images. As seen in confocal images, transmigration of CD4^+^ T cells was decreased in hypoxic organoids compared to normoxic control, *p* = 0.0008 (two-tailed paired Student’s *t*-test). Transmigration significantly decreased in control normoxic organoids, *p* = 0.0003, but did not decrease with anti-ICAM1 blocking in OGD organoids, one-way ANOVA with Dunnett’s test. Graph points represent individual flow cytometry reading from 8 pooled organoids. Data driven from six biological replicates. All data are presented as mean ± SD. Scale bar (**A**) 200 μm. Level of significance is 0.05, with symbols * for *p* < 0.05, *** for *p* < 0.001, and ns for not significant (>0.05).

Transmigration experiments were repeated with 12 h OGD (hypoxia) organoids. Immune cells were allowed to migrate for 6 h before the end of OGD with or without anti-ICAM1 blocking. Blocking with anti-ICAM1 was conducted for one hour before the start of the transmigration experiment. Confocal images were captured for organoids by the end of the 6 h (Figure 10A–C). Similar images were captured for normoxic control after transmigration with and without anti-ICAM1 treatment and isotype control was included as well (Figure 10D–F). Isotype control was used to exclude Fc receptor nonspecific binding to cell adhesion molecules and nonspecific blockade of transmigration.

ImageJ analysis was conducted to measure the number of immune cells from confocal images (Figure 10G). Isotype controls did not show significant differences in the number of transmigrated cells for both normoxic and OGD organoids. There was a significant decrease in the transmigration of T cells under OGD compared with normoxic controls using paired Student’s *t*-test (*p* = 0.0009, Figure 10G). Anti-ICAM1 antibody caused significant reduction in transmigration in normoxic organoids *p* = 0.013 compared to unblocked normoxic organoids, but it did not decrease transmigration in OGD organoids. To confirm the changes in immune cell transmigration, flow cytometry analysis was conducted in dissociated organoids to quantify immune cells (Figure 10H). Comparable results were confirmed with transmigration significantly reduced in OGD organoids compared to normoxic ones *p* = 0.0008. Isotype control antibodies did not reveal differences from unblocked normoxic or OGD organoids. Similar to confocal analysis, flow cytometry showed significant reduction in transmigration in normoxic organoids *p* = 0.0003, but not OGD organoids. Flow cytometry histograms are shown in Supplementary Figure S7.

### 3.11. Increased Transmigration of Activated CD4^+^ T Cells Across the BBB with Reperfusion and Reduction with Anti-Cell Adhesion Molecule Antibodies

**Figure 11:**
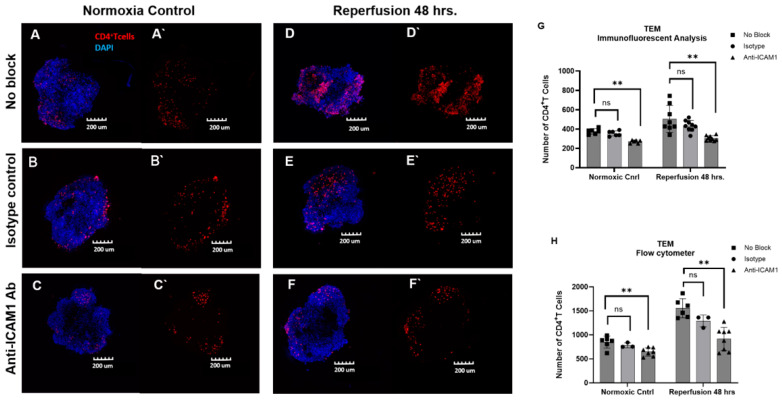
**Reperfusion impacts activated CD4^+^ T cell transmigration into BBB organoids**. Activated T-cells were stained with red PKH26 cell tracker and incubated with the control normoxic organoids and organoids reperfused for 48 h. For blocking assay, the anti-ICAM1 antibody was incubated with the organoids at 1 h prior to the transmigration experiment. (**A**–**C**) Normoxic organoids merged images for dapi (blue) and immune cells (red), and (**A`**–**C`**) images for red channel only. (**D**–**F**) Merged images for organoids after reperfusion for 48 h, and (**D`**–**F`**) images for red channel only. Organoids were imaged by confocal laser scanning microscopy and images represented as Z-stack projections of serial slices. (**G**) Transendothelia migration (TEM) of activated CD4^+^ T cells across the BBB was significantly higher after reperfusion for 48 h compared to transmigration in normoxic control *p* = 0.025 (two-tailed paired Student’s *t*-test). Transmigration of activated T-cells was significantly reduced with anti-ICAM1 antibodies in both normoxic organoids and after reperfusion for 48 h compared to transmigration without blocking antibodies, *p* = 0.002 and *p* = 0.001, respectively. One-way ANOVA with Dunnett’s test was used for statistical significance. Individual points represent individual organoids from three biological replicates. (**H**) Flow cytometry analysis of the number of CD4^+^ T cells in organoids confirmed changes detected by immunofluorescent images after reperfusion for 48 h. Transmigration of CD4^+^ T cells was increased in organoids reperfused for 48 h compared to normoxic controls, *p* = 0.0007 (two-tailed paired student *T*-test). Transmigration decreased with anti-ICAM1 blocking antibodies in both normoxic and reperfused organoids, *p* = 0.006 and *p* = 0.002 respectively. Individual points represent individual flow cytometry reading from 8 pooled organoids. Data are driven from six biological replicates. Graphs are presented as mean ± SD. Scale bar (**A**) 200 **μm**. Level of significance is 0.05, with symbols ** for *p* < 0.01, and ns for not significant (>0.05).

Since the highest expression of cell adhesion molecules was achieved after 48 h of reperfusion, transmigration experiments were repeated with reperfusion for 48 h. Transmigration was allowed, with or without ICAM1 blocking, for 6 h before the end of 48 h transmigration time point. Blocking with Anti-ICAM1 was conducted for one hour before the start of transmigration experiment. Confocal images where captured for organoids by the end of the 6 h (Figure 11A–C). Matching normoxic controls were also images with and without ICAM1 blocking and isotype control included (Figure 11D–F).

ImageJ analysis for transmigration showed significant increase in transmigration with 48 h reperfusion *p* = 0.025 comparing it to matched normoxic control using Student’s *t*-test. Anti-ICAM1 blocking significantly decreased transmigration in both 48 h reperfusion *p* = 0.001 and its normoxic matched control *p* = 0.002. Isotype controls did not show significant differences (Figure 11G). To confirm changes, flow cytometry analysis was conducted showing the same significant increase in transmigration of immune cells with reperfusion for 48 h compared to normoxic control *p* = 0.0007. Anti-ICAM1 antibodies decreased transmigration with 48 h reperfusion *p* = 0.002 and normoxic control *p* = 0.006 and no changes detected in transmigration in isotype controls (Figure 11H). Similar to transmigration experiment after inflammatory cytokine treatment, the model succeeded in reflecting changes in immune cell transmigration in a more clinically relevant model that facilitates the establishment of pre-clinical in vitro human model to study effective therapeutic interventions in different clinical conditions.

## 4. Discussion

Immune dysregulation is involved in the pathophysiology of many brain disorders, including multiple sclerosis, ischemia–reperfusion injury, traumatic brain injury, neurodegenerative diseases, epilepsy, and even psychiatric disorders [35]. It was previously thought that the CNS is an immune-privileged organ with no connection to the peripheral immune system, but this concept is now evolving, especially with the discovery of the draining lymphatic system in the brain [36]. The availability of in vitro BBB models enables investigations of the BBB–immune system interface and allows the testing of different anti-inflammatory and immunomodulatory strategies for treatment. Most of the studies investigating immune cell interaction at the BBB used 2D cell culture models, or transwell models that fail to recapitulate the cellular complexity of the human NVU. The presence of different CNS cells is essential for recapitulating BBB properties [6,37] and responding to neuroinflammatory injury and ischemia–reperfusion injury (IRI) [38]. Furthermore, if non-human cell types are used, they do not precisely recapitulate human responses. The use of physiological relevant human model that accounts for the complexity of human systems will accelerate the transfer of preclinical results to the clinic [39,40].

We previously established a human 3D multicellular BBB model formed by self-assembly of BMVECs and human brain vascular pericytes around a core of neuroglial cells [20,21]. In this model, all cells are human in origin and are in direct contact with each other. Cell ratios were based on successful assembly and integrity of the model taking into consideration averaged ratios of different cell types from various parts of the brain [41,42,43]. The potential of the model to recapitulate various aspects of BBB was also tested in detail in that previous work.

The aim of the current study was to validate this human 3D BBB model to investigate the transmigration of immune cells and determine differences between normal and inflammatory conditions. These data demonstrate the model’s potential for testing novel anti-cell adhesion molecules.

To model neuroimmune interactions in vitro, 3D BBB organoids were treated with inflammatory cytokines, and their effects on the BBB were measured. The dose of 10 ng/mL was chosen, based on previous research work, where 10 ng/mL was found to be the lowest concentration to achieve changes in tight junction expression [44]. Other work that used that same dose to induce microvascular endothelial cells hyperpermeability [45]. Furthermore, neuroinflammation is the major contributor to the pathophysiology of IRI in the brain as many cells contribute to the release of inflammatory cytokines, including vascular endothelial cells and microglia, which are brain-resident immune cells [46,47,48]. Sharing a common inflammatory element, we also applied an oxygen–glucose deprivation–reoxygenation/reperfusion (OGD-R) model to mimic IRI using the in vitro 3D BBB organoids [49]. To our knowledge, this is the first in vitro 3D BBB model used to investigate immune cell transmigration, neuroinflammation, and OGD-R.

The development of hypoxia in the OGD model was confirmed by staining with Image-iT™ Green. The expression of the transcription factor HIF-1α, which responds to hypoxia, was upregulated in this model after 12 h of hypoxia exposure. HIF-1α upregulation has also been previously reported with in vitro and in vivo ischemia models [50].

ATP production significantly decreased with acute hypoxia in the 3D BBB model and continued to decrease even with reperfusion. Decreased ATP production could be due in part to a shift to anaerobic respiration as a result of deficiency in the oxygen supply. Cell death due to the release of inflammatory cytokines could also contribute to ATP reduction [51,52,53].

Inflammatory cytokines such as TNF-α and IFN-γ are known to induce apoptosis [54,55]. We demonstrated that inflammatory cytokines induced cell death in our BBB organoids. Similarly, acute ischemia and subsequent reperfusion reportedly activate neuroinflammatory pathways that lead to cell death [46]. Cell death was detected in the OGD-R model and increased significantly with hypoxia but increased further with reperfusion after 72 h. Cytokines produced by native brain cells, mitochondrial disruption, and energy failure or HIF-1α-induced apoptotic pathways could all contribute to this second phase of increased cell death. Cell death and apoptosis can result from both caspase-dependent (ATP-dependent) and caspase-independent pathways in the ischemic organoid core [56]. To validate the element of inflammation in OGD-R model, TNF-α levels were assessed. Increased levels of TNF-α were detected under hypoxia and continued to increase later with reperfusion, with a peak occurring after 72 h. During ischemia and after reperfusion, many cells, such as astrocytes, microglia, and immune cells, contribute to the production of different inflammatory cytokines [57,58]. It has been reported that TNF-α mRNA expression increases just three hours after the start of hypoxia, and protein levels continue to increase up to 5 days after stroke onset [59]. TNF-α was also found to increase the production of MMP-9 through a cyclooxygenase-1-mediated process that contributes to BBB disruption [60].

MMP-9 is used as a clinical marker and predictor of the severity and prognosis of ischemic stroke. Its levels correlate with poor neurological outcomes and the development of hemorrhagic transformation after rtPA therapy. MMP-9 secretion is reported to increase a few days after an ischemic event and contributes to tight junction and basement membrane degradation [61,62,63,64]. In the current model, MMP-9 expression showed a trend towards increasing after 48 h reperfusion even though not statically significant. However, MMP-9 expression was not increased in other time points in our model. Lack of immune cells throughout the whole experiment might have contributed to this. The mild increase after reperfusion for 48 h might be attributed to other brain such as astrocytes and microglia which have also been reported to be a source of MMP-9 [65,66].

Increased reactive oxygen species production is associated with mitochondrial dysfunction, electron imbalance, and inflammation [67]. We were able to demonstrate that ROS production increased with hypoxia and remained above baseline during reperfusion. ROS production showed another significant peak with reperfusion at 72 h. Although additional investigations are needed to detail the specific ROS moieties and the cells of origin in this 3D BBB model, targeting the production of ROS remains important for treating IRI, as ROS production aggravates brain damage by activating other injury pathways, such as oxidative DNA damage and protein kinase activation [68].

The BBB is characterized by tight junctions [69]. Small molecules of various molecular weights, such as dextrans, can be used to detect changes in permeability in 3D models [37]. In our model, the 70 kDa dextran permeability assay revealed an increase in permeability with exposure to both cytokines, although the difference from controls was greater with TNF-α than with IFN-γ. This finding reflects the disruption of the paracellular junctions in the BBB induced by both cytokines. TNF-α has been consistently reported to increase BBB permeability [70,71], but contradictory effects of IFN-γ on BBB permeability have been shown, with reports demonstrating a permeability increase [72,73,74] and others showing a decrease. This paradox in the IFN-γ effect may be attributed in part to the different models used and the nonhuman cells used [75]. We demonstrated that BBB permeability increases during and after ischemia, with a second peak occurring after 72 h of reperfusion. This coincides with the increase in cell death, and TNF-α secretion. Our model suggests that BBB permeability evolves over time after injury.

Occludin is a transmembrane protein crucial for the formation of the BBB [76]. We showed that both TNF-α and IFN-γ decreased occludin expression after exposure for 24 h. Comparable results were reported in other studies [76,77]. Other reports have demonstrated that IFN-γ could have a different effect on occludin by upregulating its expression or stabilizing it [78,79]. To further clarify this point, different doses and exposure times should be compared. Claudin-12 expression at the BBB has been confirmed [34,80]. Here, we show that the expression of claudin-12 was detectable in our model and that its expression was responsive to exposure to both cytokines, as shown by decreased mRNA and protein levels. Although the role of claudin-12 in disorders of the BBB has yet to be fully investigated, these data indicate a potential link between inflammation and altered claudin-12.

After hypoxia and reperfusion for 72 h, the expression of tight junctions significantly decreased, which also highlights the impact of IRI on the transcription of tight junctions. Tight junction degradation and TJ protein relocation after ischemia–reperfusion injury has been well reported [81,82]. Our results reflect changes in mRNA expression, and future characterization of protein expression and posttranslational modifications might provide more insights into the functional state of these proteins following their mRNA expression.

Inflammatory cytokines have long been implicated in the upregulation of cell adhesion molecules and in modulating the transmigration of immune cells across the BBB in neuroinflammatory and neuroimmunological disorders. Here, we show the upregulation of two important adhesion molecules, VCAM1 and ICAM1, in response to TNF-α and IFN-γ in accordance with previous studies [83,84]. Furthermore, cell adhesion molecules were upregulated with reperfusion and peaked after 48 h of reperfusion. Similar upregulation of cell adhesion molecules has been previously demonstrated in cerebral ischemia models [85,86].

Even though protein analysis by ELISA showed similar trends for measured markers, it was not as significant as the RNA levels. This discrepancy between RNA and protein is common since changes in mRNA precede protein synthesis changes, and the synthesis of proteins are governed by many other regulatory processes such as posttranslational regulation or protein turnover rates. Also, qPCR and ELISA have different sensitivity thresholds.

The upregulation of adhesion molecules was reflected by the significant increase in the transmigration of immune cells across the BBB in our 3D model. Transmigration was significantly blocked with an anti-ICAM1 antibody after reperfusion for 48 h. No reduction was observed with anti-ICAM1 after OGD, which warrants more investigations. The shutdown of cellular machinery during ischemia resulting in dysregulation or downregulation of several proteins including cell adhesion molecules might have contributed to this finding. Furthermore, under different pathological conditions alternative transmigration pathways could be triggered that are ICAM1-independent. Decreased T cell viability and movement could also contribute to this.

Using anti-ICAM1-blocking antibodies in both murine [87,88] and rabbit [89,90] models, decreased immune cell recruitment and the resulting infarct size was detected. Despite promising results in in vivo models and the establishment of safe treatments [91], human clinical trials have shown that Enlimomab (anti-ICAM1) is not effective as a treatment in acute stroke trials [92]. This might arise from the gaps that exist between preclinical animal models or in vitro models that utilize animal cells and less complex systems that cannot precisely recapitulate the true human responses in vivo. Furthermore, the only anti-cell adhesion molecule antibody that has been translated into clinical practice is anti-alpha 4 integrin (Natalizumab). Although other adhesion molecules, including PECAM1, ALCAM, and CerCAM, have been discovered and their role in immune cell transmigration has been elucidated [93,94,95], none have yet succeeded in reaching clinical application.

There are some limitations to our current model and interpretation of the data. With the tremendous advancements in in vitro BBB modeling, it is challenging to conduct a thorough comparison of the results concluded from different studies across BBB in vitro models that differ in the source of cells used, the number of cells used in coculture, and the structural design of the model [96]. It is still necessary to investigate the response of different cell adhesion molecules to IRI and elucidate their therapeutic potential in decreasing immune cell recruitment after ischemic stroke, especially pathogenic immune cells such as IL-17-producing T cells [97]. The investigation of anti-cell adhesion molecule therapies would benefit from investigating the treatments with different doses and for different durations to fully capture significant therapeutic effects. With respect to mRNA and protein expression data, it is common for those endpoints to not correlate in amplitude or timing, based on many levels of transcriptional, translational, and post-translational regulation. Also, the inclusion of microfluidics to the model may provide flow-induced forces that affect the maturation of BBB properties, immune cell transmigration and hence affect the outcome of transmigration experiments [98,99].

The current model represents a multicellular platform to investigate BBB properties and BBB-related responses under disease conditions. However, this model could benefit from detailed studies to highlight the contribution of each cell type to the responses.

Moreover, as immune cell transmigration is not dependent on one factor such as cell adhesion molecules upregulation, the current model could be expanded to further study the contribution of other molecules such as selectins, chemokines and other regulatory mechanisms that affect the process of immune cell transmigration under normal and disease conditions.

In our current work, the modeled interactions between immune cells and the 3D human BBB organoid represent an opportunity to study potential therapeutic targets that could ameliorate outcomes in inflammatory conditions and in ischemic patients prone to neurological complications associated with reperfusion. The functionality of our BBB model offers many opportunities to investigate various categories of drugs, such as anti-cell adhesion molecule antibodies, anti-inflammatories, or drugs that target microglia, protect the BBB, and reduce ROS.

## 5. Conclusions

Studying neuroimmune interactions is becoming increasingly important, with increasing evidence accumulating on the contribution of the immune system to neurological diseases previously thought to be only degenerative in nature and extending even to psychiatric diseases.

BBB transwell models have been used to study the BBB and its interactions with different biological and chemical exposures. Most BBB models involve monolayers of brain endothelium that are mainly of nonhuman origin, and some of them involve cocultures of only pericytes or astrocytes. As evidence continues to accrue that all components of the NVU participate in either the formation or integrity of the BBB, the search for a more physiological model to recapitulate interactions at the surface of the BBB is important.

As shown in this report and in previous publications from our group, our 3D BBB model is composed of all six major human brain cell types, in a similar proportion to that noted in human brains and resembles the natural NVU in many key structural and functional aspects. Further characterization of molecules expressed at the barrier surface and their function, along with investigations of the connectivity and crosstalk between different NVU elements and BMVECs will provide additional power to this model.

Investigating the transmigration of different immune cell subsets and the search for participating cell adhesion molecules in this physiological model would enhance the ability to probe other adhesion molecules for therapeutic potential.

## Data Availability

The original contributions presented in this study are included in the article/Supplementary Materials. Further inquiries can be directed to the corresponding author. The raw data supporting the conclusions of this article will be made available by the authors on request.

## Supplementary Materials

The following supporting information can be downloaded at https://www.mdpi.com/article/10.3390/cells15131173/s1, Figure S1: Cell viability analysis for cytokine treatment. Figure S2: Hypoxia induction and ROS production in OGD-R model. Figure S3: FITC-dextran permeability assay for cytokine treatment. Figure S4: Flow cytometry analysis of immune cell transmigration after cytokine treatment. Figure S5: Anti-cell adhesion molecule antibodies effect on CD4^+^ T cells transmigration in untreated organoids. Figure S6: Anti-cell adhesion molecule antibodies inhibit CD4^+^ T cell transmigration in TNF-α treated organoids. Figure S7: Flow cytometry analysis of immune cell transmigration in OGD-R model.

## Abbreviations

The following abbreviations are used in this manuscript:

BBB	Blood–Brain Barrier
BMVEC	Brain Microvascular Endothelial Cell
CNS	Central Nervous System
DAPI	4′,6-Diamidino-2-Phenylindole
DPBS	Dulbecco’s Phosphate-Buffered Saline
ELISA	Enzyme-Linked Immunosorbent Assay
FBS	Fetal Bovine Serum
HA	Human Astrocyte
HBVP	Human Brain Vascular Pericyte
HBMEC	Human Brain Microvascular Endothelial Cell
HM	Human iPSC-Derived Microglia
HO	Human iPSC-Derived Oligodendrocyte Progenitor
HN/ReN	Human Neural Progenitor Cell (ReNcell)
ICAM-1	Intercellular Adhesion Molecule-1
IFN-γ	Interferon-Gamma
IL-2	Interleukin-2
LFA-1	Lymphocyte Function–Associated Antigen-1
NVU	Neurovascular Unit
OGD	Oxygen–Glucose Deprivation
OGD-R	Oxygen–Glucose Deprivation–Reoxygenation
PBMC	Peripheral Blood Mononuclear Cell
PFA	Paraformaldehyde
qPCR	Quantitative Polymerase Chain Reaction
Rep	Reperfusion
ROS	Reactive Oxygen Species
RPMI	Roswell Park Memorial Institute Medium
TEM	Transendothelial Migration
TNF-α	Tumor Necrosis Factor-Alpha
VCAM-1	Vascular Cell Adhesion Molecule-1
VLA-4	Very Late Antigen-4
Z-stack	Optical Section Stack in Confocal Microscopy
