# Supplementary material for: Modeling Neuroimmunological Interactions at the Blood–Brain Barrier Using In Vitro 3D Human Organoids: Inflammation and Ischemia–Reperfusion Injury
Source: Cells. 2026 Jun 27;15(13):1173. doi: 10.3390/cells15131173 (PMC13360297; doi:10.3390/cells15131173)
Supplement: Supplementary file 1 [file cells-15-01173-s001.zip › cells-4330391-supplementary.pdf]

Supplementary Fig. 1

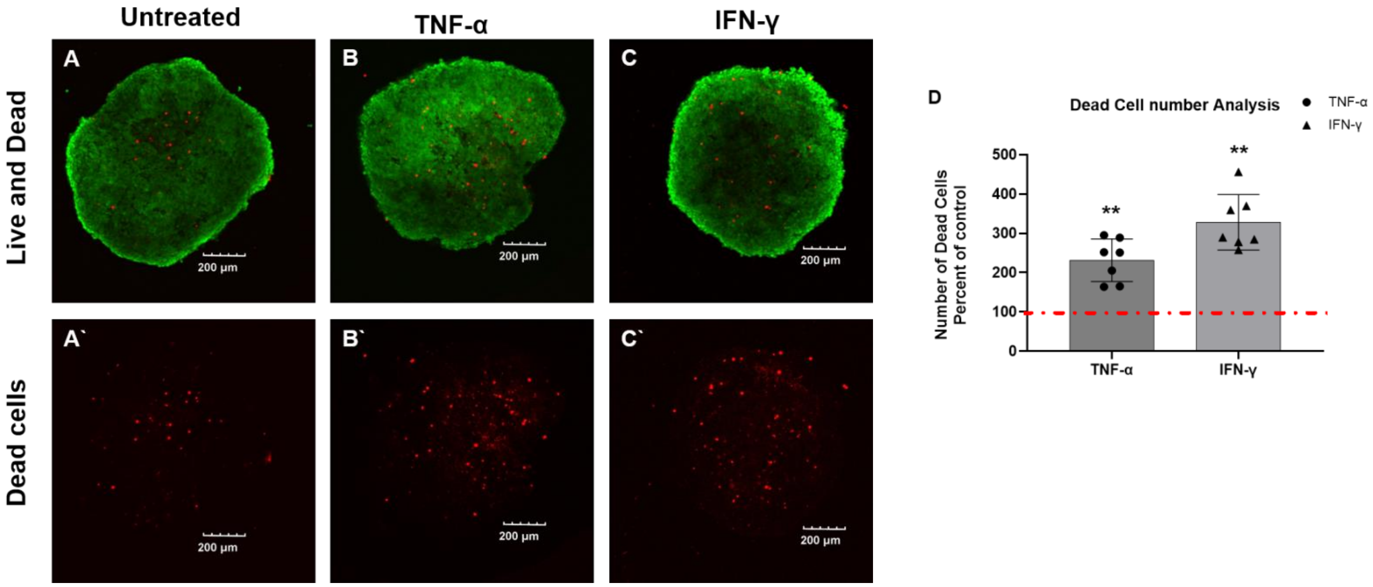

Supplementary Figure 1: Cell viability analysis for Cytokine Treatment. (**A-C**) merged and (**A`-C`**), red channel only, show organoids stained with Calcein (green-live) and ethidium bromide (red-dead) after treatment with pro-inflammatory cytokines TNF-α and IFN-γ for 24 hrs. Organoids were washed and imaged by confocal laser scanning microscopy. Images represent Z-stacks of at least 5 slices. **(D)** The number of dead cells were increased after treatment with both cytokines compared to untreated organoids, p=0.007 and p=0.008, for TNF-α and IFN-γ, respectively. Untreated controls values correspond to 100 (red dotted line). Individual points represent individual organoids from three biological replicates. Two tailed paired Student's t-test was used to calculate statistical significance from raw numbers of dead cell in each experimental group and its own time-matched untreated control organoids. Scale Bar (**A-B**) 200 um and all images were capture at the same magnification. Level of significance is 0.05, with symbols * for p < 0.05, ** for p < 0.01, *** for p < 0.001, **** for p < 0.0001, and ns for not significant(>0.05).

Supplementary Fig.2

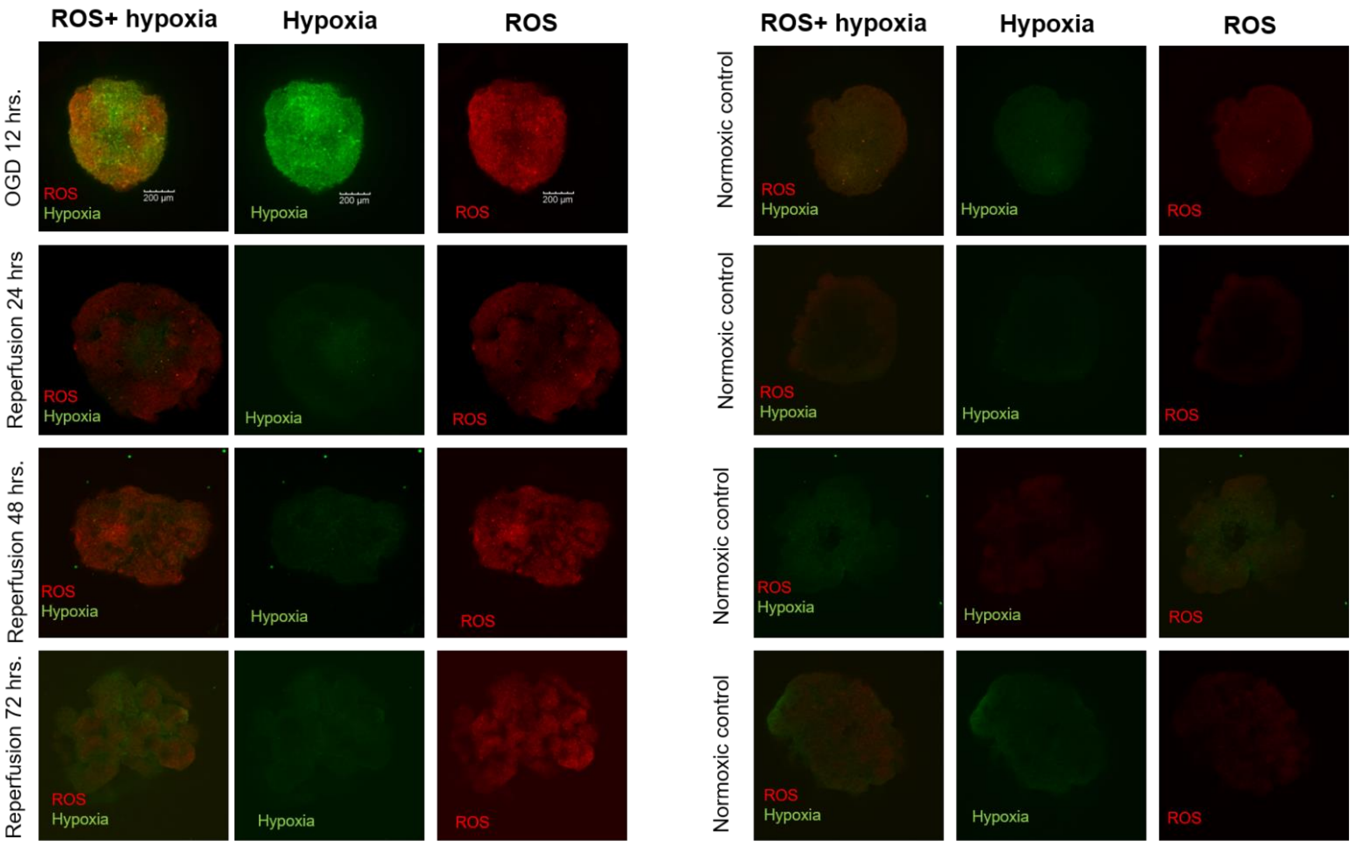

Supplementary Figure 2: Hypoxia induction and ROS production in OGD-R model. The first and third columns represent merged colors, the second and 4th column is hypoxia green fluorescence, and the third and 6th columns are cellular ROS red fluorescence. Note the increased hypoxia and ROS production after OGD that decreased with reperfusion (columns 1-3). Time matched normoxic controls showed little hypoxia or ROS (columns 4-6). Images were captured using laser confocal scanning microscopy. At least 5 Z stacks were merged into Z projection for each Image. Scale Bar 200 um and all images captured at same magnification.

Supplementary Fig. 3

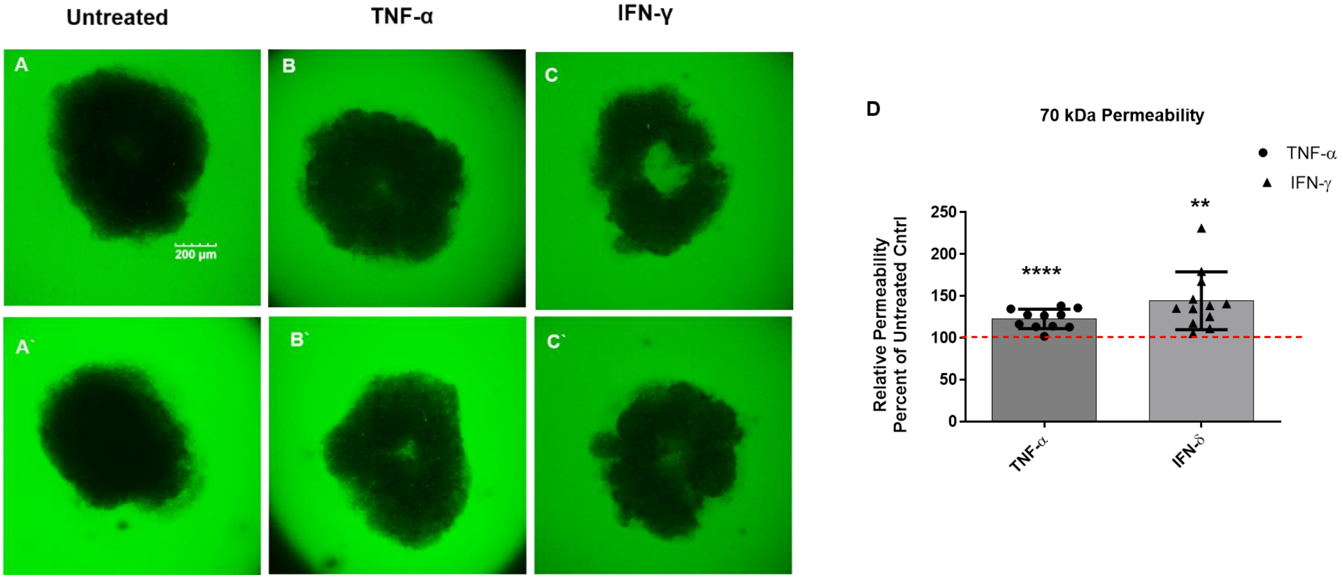

Supplementary Figure 3: FITC-dextran permeability assay for Cytokine Treatment. **(A-C)** shows confocal images for organoids incubated with 70 kDa FITC-dextrans (50 ug/ml) for 30 minutes after 24 hrs. of treatment with cytokines. **(D)** The intensity of green fluorescence inside the organoids was measured using ImageJ and relative permeability of each organoid was calculated as a percent of relative permeability (inside to outside green ratios) of its own matched untreated control, normalized to the untreated group as 100% (red dotted line). Permeability was increased above baseline in TNF-α, $p<0.0001$ and IFN-γ, $p=0.002$, two tailed paired Student's t-test. Individual points represent individual organoids from three biological replicates. Scale bar 200 um (A) and all images captured at same magnification. Level of significance is 0.05, with symbols * for $p < 0.05$, ** for $p < 0.01$, *** for $p < 0.001$, **** for $p < 0.0001$, and ns for not significant(>0.05).

Supplementary Fig. 4

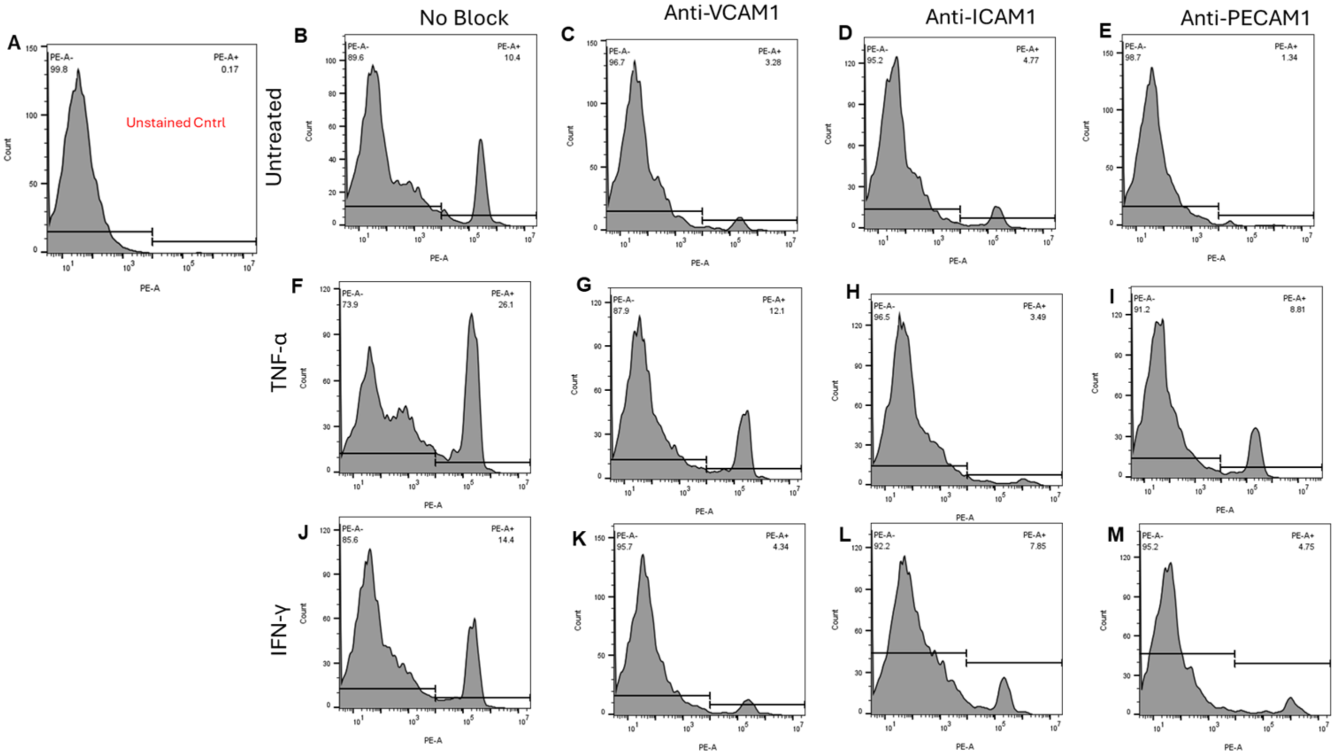

Supplementary Figure 4: Flow cytometry analysis of immune cell transmigration after cytokine treatment. Organoids were incubated with PKH26 stained $CD4^{+}$ T cells and dissociated after transmigration experiments. Cells were fixed with 4%PFA and the number of positive stained PKH26 T cells (right hand peak in panels B-M) determined by flow cytometry counting 10,000 cells from the dissociated organoids. Transmigration of $CD4^{+}$ T cells significantly increased with both TNF-α (F) and IFN-γ (J) treatment (see text for statistics). Anti-cell adhesion molecule antibodies (anti-VCAM1, anti-ICAM1 and anti-PECAM1) significantly decreased the number of transmigrating cells across all groups.

Supplementary Fig. 5

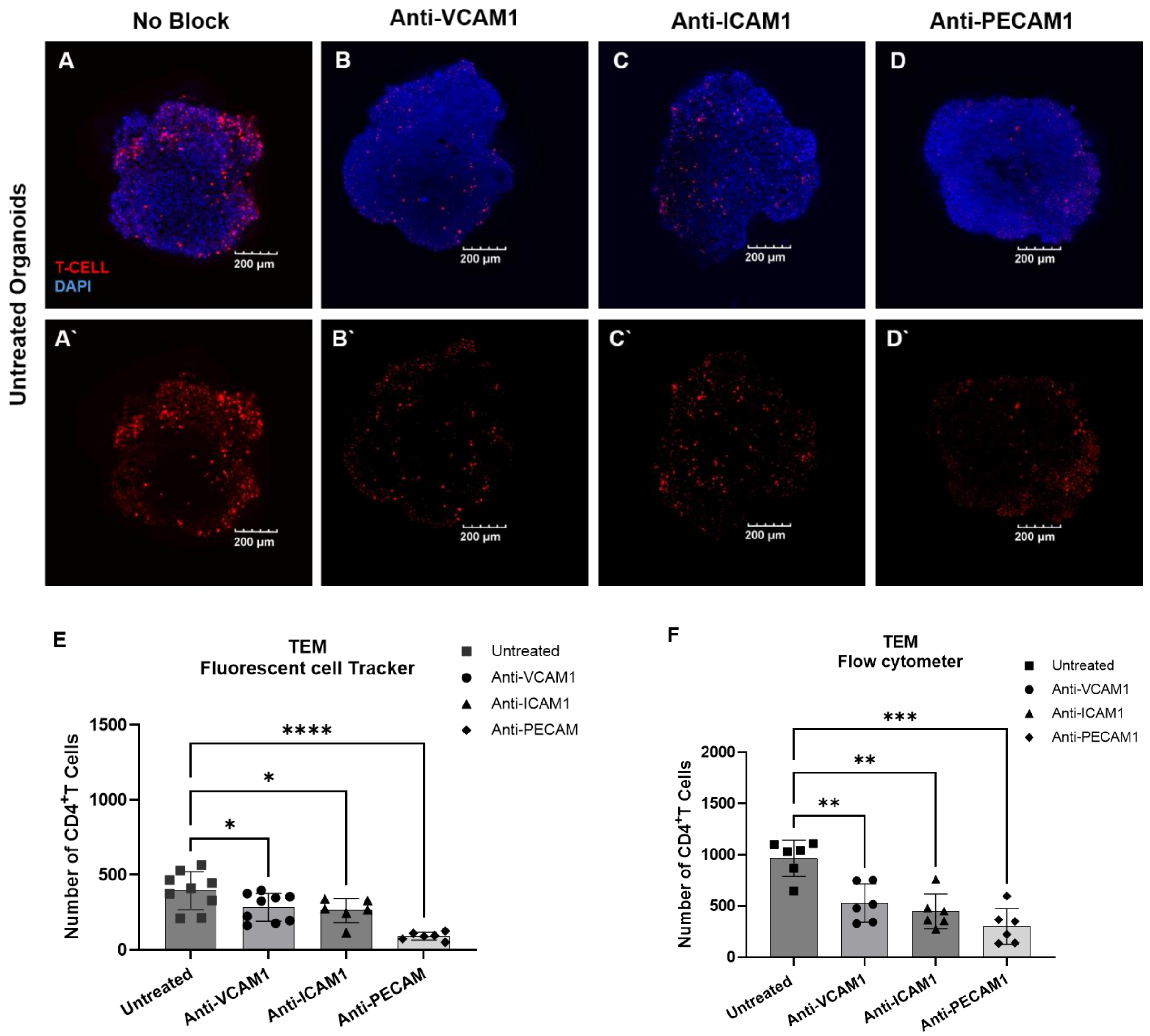

Supplementary Figure 5: Anti-cell adhesion antibodies effect on CD4+ T cells transmigration in Untreated Organoids. Activated T-cells were stained with red PKH26 cell tracker and incubated with untreated organoids. **(A-D)** Merged images for dapi (blue) and immune cells (red). **(A`-D`)** Images show immune cells in red only channel. Organoids were imaged by confocal laser scanning microscopy and images represented as Z-stack projections of serial slices. **(E)** Analysis shows that transmigration of activated $CD4^+$ T cells across the BBB was significantly lower after treatment with three different anti-cell adhesion antibodies; anti-VCAM1, anti-ICAM1 and anti-PECAM1, p= 0.037, 0.014 and <0.0001, respectively. One-way ANOVA with Dunnett's test was used to calculate statistical significance. Individual points represent individual organoids from three biological replicates. **(F)** Flow cytometry analysis of the number of CD4+ T cells in organoids confirmed changes detected from immunofluorescent images. Similar decreases were detected with the three anti-cell adhesion antibodies with p values of 0.005, 0.005 and <0.0001 for anti-VCAM1, anti-ICAM1 and anti-PECAM1, respectively. Individual points represent individual flow cytometry readings from 8 pooled organoids (n=6). Scale bar (**a**) 200 um. Level of significance is 0.05, with symbols * for p < 0.05, ** for p < 0.01, *** for p < 0.001, **** for p < 0.0001, and ns for not significant(>0.05).

Supplementary Fig. 6

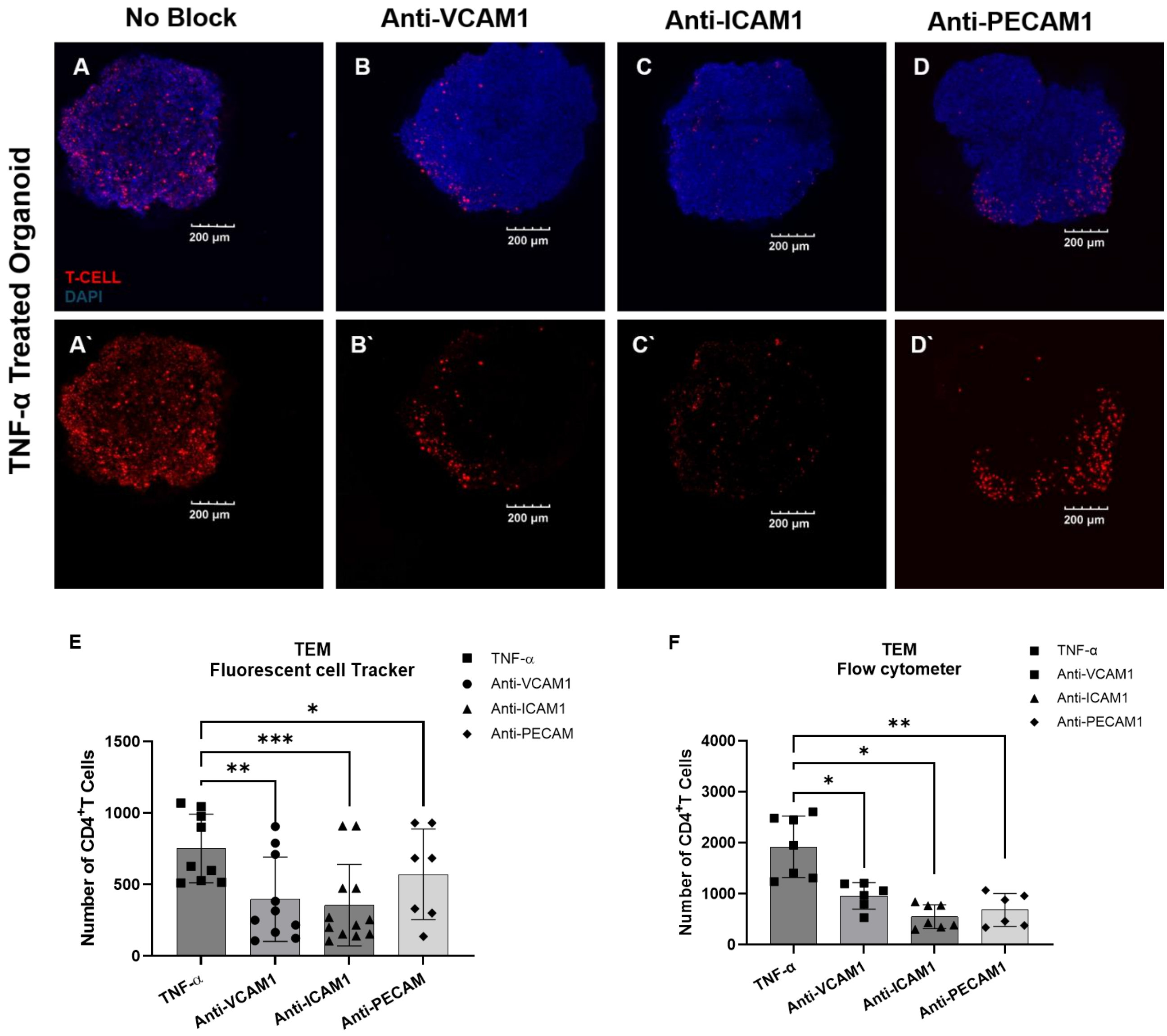

Supplementary Figure 6: Anti-cell adhesion antibodies inhibit CD4$^+$ T cell transmigration in TNF-α Treated Organoids. Activated T-cells were stained with red PKH26 cell tracker and incubated with TNF-α treated organoids. **(A-D)** Merged images for dapi (blue) and immune cells (red). **(A`-D`)** Images show immune cells in red only channel. Organoids were imaged by confocal laser scanning microscopy and images represented as Z-stack projections of serial slices. **(E)** Analysis of fluorescent confocal images shows that transmigration of activated CD4$^+$T cells across the BBB was significantly lower after treatment with three different anti-cell adhesion antibodies; anti-VCAM1, anti-ICAM1 and anti-PECAM1, p= 0.007, 0.0005 and <0.013, respectively. One-way ANOVA with Dunnett's test was used to calculate statistical significance. Individual points represent individual organoids from three biological replicates. **(F)** Flow cytometry analysis of the number of CD4$^+$ T cells in organoids was performed to confirm changes detected by confocal images. Similar decrease was detected with the three anti-cell adhesion antibodies with p values of 0.012, 0.01 and 0.002 for anti-VCAM1, anti-ICAM1 and anti-PECAM1, respectively. Individual points represent individual flow cytometry reading from 8 pooled organoids (n=6). Scale bar (**a**) 200 um. Level of significance is 0.05, with symbols * for $p < 0.05$, ** for $p < 0.01$, *** for $p < 0.001$, **** for $p < 0.0001$, and ns for not significant(>0.05).

Supplementary Fig. 7

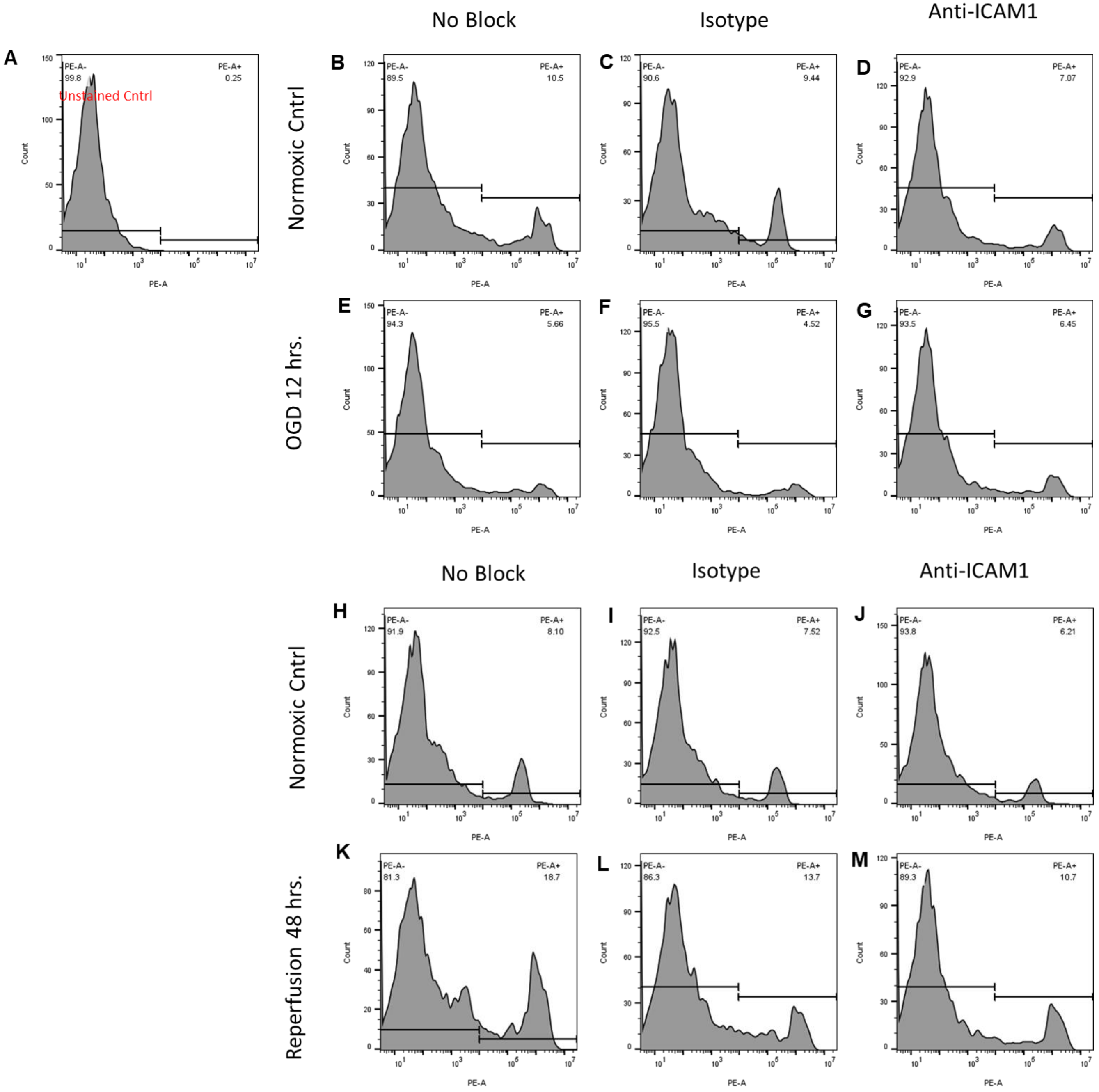

Supplementary Fig.7: Flow cytometry analysis of immune cell transmigration in OGD-R model. Organoids were incubated with PKH26 stained $CD4^+$ T cells, dissociated after transmigration experiments, cells were fixed with 4%PFA, and the number of positive stained PKH26 T cells determined by flow cytometry counting 10,000 dissociated cells per sample. Transmigration of CD4+ T cells was decreased with hypoxia (**E**) compared to normoxic control (**B**). Transmigration increased in organoids reperfused for 48 hrs. (**K**) compared to normoxic control p=0.0202 (**I**). Transmigration decreased with anti-ICAM1 blocking antibodies in both normoxic and reperfused organoids, p=0.0260 (**c**) and p=0.0324 (**l**) respectively.
